# International Imaging Practices for Sacroiliac Joint MRI Acquisition Protocols in Patients with Juvenile Spondylarthritis

**DOI:** 10.3390/jcm15176816

**Published:** 2026-09-02

**Authors:** Arthur B. Meyers, Mirkamal Tolend, Walter P. Maksymowych, Rawan Hafiz, Tarimobo M. Otobo, Jacob L. Jaremko, Robert G. W. Lambert, Nele Herregods, Marion A. J. van Rossum, Jennifer Stimec, Simone Appenzeller, Shirley Tse, Philip G. Conaghan, Lennart Jans, John A. Carrino, Eva Kirkhus, Joke Dehoorne, Pamela F. Weiss, Andrea S. Doria

**Affiliations:** 1 Cincinnati Children’s Hospital Medical Center, Department of Radiology, University of Cincinnati, Cincinnati, OH 45229, USA; 2 Department of Diagnostic & Interventional Radiology, The Hospital for Sick Children, University of Toronto, Toronto, ON M5G 0A4, Canada; mirkamal.tolend@sickkids.ca (M.T.); jennifer.stimec@sickkids.ca (J.S.); andrea.doria@sickkids.ca (A.S.D.); 3 Department of Medical Imaging, University of Toronto, Toronto, ON M5G 0A4, Canada; 4 Department of Medicine, University of Alberta, Edmonton, AB T6G 2B7, Canada; walter.maksymowych@ualberta.ca; 5 CARE Arthritis, Edmonton, AB T6G 2B7, Canada; 6 Faculty of Medicine, King Abdulaziz University, Jeddah 21589, Saudi Arabia; rhafidh@kau.edu.sa; 7 Department of Radiology, King Abdulaziz University Hospital, Jeddah 22252, Saudi Arabia; 8 Joint Department of Medical Imaging, University of Toronto, Toronto, ON M5T 1W7, Canada; tarimobo.otobo@mail.utoronto.ca; 9 Department of Radiology and Diagnostic Imaging, University of Alberta, Edmonton, AB T6G 2T4, Canada; jjaremko@ualberta.ca (J.L.J.); rlambert@ualberta.ca (R.G.W.L.); 10 Department of Radiology and Nuclear Medicine, Ghent University Hospital, 9000 Ghent, Belgium; nele_herregods@telenet.be (N.H.); lennart.jans@ugent.be (L.J.); 11 Department of Pediatrics, Emma Children’s Hospital, Academic Medical Center, 1105 Amsterdam, The Netherlands; m.a.vanrossum@amc.nl; 12 Amsterdam Rheumatology and Immunology Center, 1007 Amsterdam, The Netherlands; 13 Autoimmunity Lab, School of Medical Science, Universidade Estadual de Campinas, Campinas 13083-887, Brazil; appenzellersimone@yahoo.com; 14 Department of Orthopedics, Rheumatology and Traumatology, School of Medical Science, Universidade Estadual de Campinas, Campinas 13083-894, Brazil; 15 Department of Paediatrics, Division of Rheumatology, The Hospital for Sick Children (SickKids), University of Toronto, Toronto, ON M5G 1X8, Canada; shirley.tse@sickkids.ca; 16 Leeds Institute of Rheumatic and Musculoskeletal Medicine, University of Leeds, Leeds LS9 7TF, UK; p.conaghan@leeds.ac.uk; 17 NIHR Leeds Biomedical Research Centre, Leeds LS7 4SA, UK; 18 Department of Radiology and Imaging, Hospital for Special Surgery, New York, NY 10021, USA; carrinoj@hss.edu; 19 Division of Radiology and Nuclear Medicine, Oslo University Hospital, Nydalen, P.O. Box 4956, 0424 Oslo, Norway; ekirkhus@ous-hf.no; 20 Department of Internal Medicine and Pediatrics, Ghent University Hospital, 9000 Ghent, Belgium; joke.dehoorne@uzgent.be; 21 Department of Pediatric Rheumatology, Ghent University Hospital, 9000 Ghent, Belgium; 22 European Reference Network for Rare Immunodeficiency, Autoinflammatory and Autoimmune Diseases, The Ghent University Hospital, 9000 Ghent, Belgium; 23 Department of Pediatrics and Epidemiology, Perelman School of Medicine, University of Pennsylvania, Philadelphia, PA 19104, USA; weisspa@chop.edu; 24 Division of Rheumatology and Clinical Futures, Children’s Hospital of Philadelphia, Philadelphia, PA 19146, USA

**Keywords:** pediatric ankylosing spondylitis, spondyloarthropathy, sacroiliitis, magnetic resonance imaging

## Abstract

**Background/Objective**: International recommendations exist for MRI evaluation of sacroiliac joints (SIJs) in adult spondylarthritis (SpA) and other joints in juvenile (J)SpA. However, consensus recommendations for MRI acquisition protocols for SIJs in JSpA are lacking. Our survey gathered information on current imaging practices on SIJ MRI and expert opinions about imaging protocols. **Methods**: A survey was sent to an international group of pediatric radiologists and rheumatologists. Participants were asked about practices regarding sequences/planes, use of contrast, and preferred sequences/planes for evaluating individual lesions. **Results**: There were 38 unique survey respondents (North America, *n* = 19; South America, *n* = 1; Europe, *n* = 12; Asia, *n* = 2; Australia, *n* = 2; unknown = 2) from 32 institutions with a median of 15 (range 3–32) years of experience. Most (93%) institutions use the 2nd sacral vertebral body posterior cortex to plan coronal oblique sequences; most perform these as non-fat-suppressed T1W and fluid-sensitive sequences for evaluation of damage and inflammation, respectively. Among the 84% of institutions performing small field-of-view imaging, all include a coronal oblique plane, and 78% also do an axial oblique plane. Of all institutions, 22% utilize gradient echo (GRE) sequences. Most respondents (79%) did not consider contrast necessary for evaluating SIJs. **Conclusions**: Areas of agreement for a consensus MRI protocol for SIJ evaluation in JSpA include using a non-contrast-enhanced protocol and the S2 vertebral body posterior cortex to plan coronal oblique sequences. Further international consensus is required concerning the optimal scan plane to prescribe for axial sequences and the incorporation of an erosion-specific sequence into standard protocols.

## 1. Introduction

MRI is widely considered the reference standard imaging modality for the overall assessment of the sacroiliac joint (SIJ) in pediatric patients with juvenile spondylarthritis (JSpA). Recently, an international group of experts serving as members of the Outcomes Measures in Rheumatology and Clinical Trials (OMERACT) in the juvenile idiopathic arthritis MRI (JAMRIS) SIJ working group developed standardized definitions for MRI assessment of SIJs in JSpA [1]. Subsequently, they reported on the reliability and effect of calibration on interpreters’ reliability when using the juvenile idiopathic arthritis MRI-SIJ (JAMRIS-SIJ) scoring system [2,3]. The OMERACT JAMRIS-SIJ scoring system encompasses two domains, one focused on inflammation and the other on structural/damage of the SIJ [1].

In 2020, the European Society of Musculoskeletal Radiology (ESSR) arthritis subcommittee and the European Society of Paediatric Radiology (ESPR) musculoskeletal imaging task force published an expert opinion article that included a consensus protocol from their group, which included three recommended sequences and two optional sequences [4]. The recommended sequences are a T2-weighted (T2W) fat-suppressed (FS) or short tau inversion recovery (STIR) coronal oblique, a T1-weighted (T1W) coronal oblique, and a T2W FS or STIR axial oblique [4]. Optional sequences in that protocol were a post-contrast T1W FS coronal oblique and a post-contrast T1W FS axial oblique [4]. In this protocol, axial images are oriented transverse to the SIJs and perpendicular to the coronal oblique plane.

Later in 2024, members of the SpondyloArthritis International Society (ASAS) and the SpondyloArthritis Research and Treatment Network (SPARTAN) published an international consensus MRI protocol for evaluating adult SIJs, which was adopted by the European Society of Radiology (ESR) and European Society of Musculoskeletal Radiology (ESSR) [5,6]. The authors of this consensus acknowledge that while the principles and sequences should have some degree of application to children, they chose not to make specific recommendations regarding pediatric SIJ protocols [6]. These authors cited a number of reasons for abstaining from making pediatric-specific recommendations primarily related to the greater difficulty in interpreting pediatric SIJ MRI. The increased difficulty in interpreting pediatric SIJ MRI occurs due to changes in the developing pediatric skeleton throughout childhood, which lead to significant age- and sex-related differences in the imaging appearance at the SIJs [7,8]. These changes are related to normal developmental features such as the presence of unossified cartilage and ossification centers, increased T2 signal in unossified cartilage and in newly formed subchondral bone, as well as increased presence of hematopoietic red marrow in younger children, which undergoes conversion to yellow marrow throughout childhood [7].

Institutions vary in their SIJ MRI acquisition protocols, and no international consensus exists concerning a standard SIJ MRI protocol for assessment of JSpA. However, international consensus MRI protocols have been developed for other joints in juvenile idiopathic arthritis (JIA) [9]. There is a recognized critical need for an international consensus protocol to guide institutions in the necessary sequences and imaging planes required to detect pathology and to allow for better inter-institutional comparison of results from clinical research in JSpA. Areas that need to be addressed are recommended sequences and planes of acquisition. Whereas both the ESSR-ESPR and ASAS-SPARTAN recommended protocols include coronal oblique T1W and T2W FS or STIR sequences, the ASAS-SPARTAN adult protocol also recommends a coronal oblique thin-slice 3D gradient echo (GRE) sequence, which is not included in the European pediatric recommendation [4,6]. Furthermore, both recommendations prescribe axial sequences oriented perpendicular to the coronal oblique plane [4,6]. However, some pediatric institutions prescribe the axial plane in relation to the longitudinal axis of the body to evaluate the remainder of the pelvis and the hip joints in a more standard plane given the high incidence of hip joint pathology in children with JSpA [10]. The most recent ESSR recommendation does state that the hips should be included in children with JSpA but does not specify the orientation of the axial acquisition required for this purpose [5]. Also, whether situations exist when post-contrast sequences should be added to the MR protocol for JSpA remains an open question [4].

In this study, we surveyed an international group of experts regarding the interpretation of SIJ MRI in pediatric patients with JSpA to document current international practices, identifying areas of agreement and disagreement. The present study is a descriptive survey of institutional practice and expert opinion and does not itself establish consensus recommendations. Our goal is to inform the future development of an international consensus MRI protocol for assessment of SIJs in children and adolescents with JSpA.

## 2. Materials and Methods

Members of the OMERACT JAMRIS working group developed an anonymous multipart REDCap survey to be sent to an international group of radiologists and rheumatologists. This survey (Supplementary File S1) was approved by the Research Ethics Board at The Hospital for Sick Children in Toronto, Canada. The demographics portion of the survey asked questions regarding participants’ institution, specialty, and years of experience evaluating SIJ MRI. Part 1 asked about specific sequences and imaging planes used at their home institutions. Part 2 asked a series of general imaging questions, including the use and perceived utility of contrast administration, landmarks used for orientation of the coronal oblique imaging plane, the MRI sequences that the participants consider essential and those that they consider optional, order of sequences, technical parameters (e.g., magnet strength, coil type, etc.), and demographics of patient populations at their institutions. Part 3 asked questions concerning the most appropriate sequences to evaluate the set of 12 items of the OMERACT JAMRIS-SIJ scoring system, encompassing both the inflammatory and damage domains. The survey went through multiple iterations, which were reviewed and approved by members of the OMERACT JAMRIS working group.

The survey was emailed to a list of 89 radiologists and rheumatologists across North America, Europe, Asia, and Australia. A purposive/convenience recruitment strategy was utilized. The email list was generated from members of various national and international radiology and rheumatology professional societies (e.g., the Society of Pediatric Radiology musculoskeletal committee, the arthritis and pediatric subcommittees of the European Society of Radiology, the Australian and New Zealand Society for Paediatric Radiology, and the Asian and Oceanic Society for Paediatric Radiology).

The survey responses were analyzed descriptively, with 95% confidence intervals (CIs) obtained from the percentile bootstrap method, resampling respondents with replacement (10,000 iterations). For continuous data responses, values were reported as median and interquartile range. For multiple-response questions on sequence-plane selection, e.g., on selecting the most preferred set of imaging sequences and planes for a particular type of lesion, the results were reported in two ways. The primary analysis presented the number and proportion of respondents selecting each sequence-plane option. This conventional method provides a directly interpretable measure of endorsement, though it does not differentiate between an option that is selected as the minimally required choice by more selective respondents and one included as an ideal but optional choice selected by more inclusive respondents. Therefore, a secondary sensitivity analysis was also performed, adjusting for the multiplicity of choices per respondent (provided in the Supplementary File): each respondent’s one full vote was split equally among their selected choices for a given multiple-response question, and these part-votes were tallied to report the normalized proportion of endorsement of each sequence-plane option.

## 3. Results

### 3.1. Respondent Demographics

Thirty-eight (43%) individuals responded to the survey. Of the 36 who provided their institutional affiliation, 19 (53%) were from North America, 12 (33%) were from Europe, 2 (6%) were from Asia, 2 (6%) were from Australia, and 1 (3%) was from South America, representing 32 unique institutions. Three institutions had more than one respondent, with two to four respondents each: questions on current imaging practice were summarized by taking the most complete response from each institution; opinion-based questions included all respondents. Survey respondents had a median of 15 years (interquartile range 10–19, range 3–32) of experience interpreting SIJ MRIs. Two (6%) rheumatologists responded to the survey with the remaining respondents being radiologists (94%). The demographics of patients followed at the respondents’ institutions were as follows: 72% only evaluate children, 28% evaluate a mix of children and adults and none of the respondents’ institutions evaluate only adults (Table 1). Out of those individuals who image both adults and children in their home institution (*n* = 7), 57% did not have different protocols for children and adults.

### 3.2. Imaging Planes and Sequences

**Figure 1:**
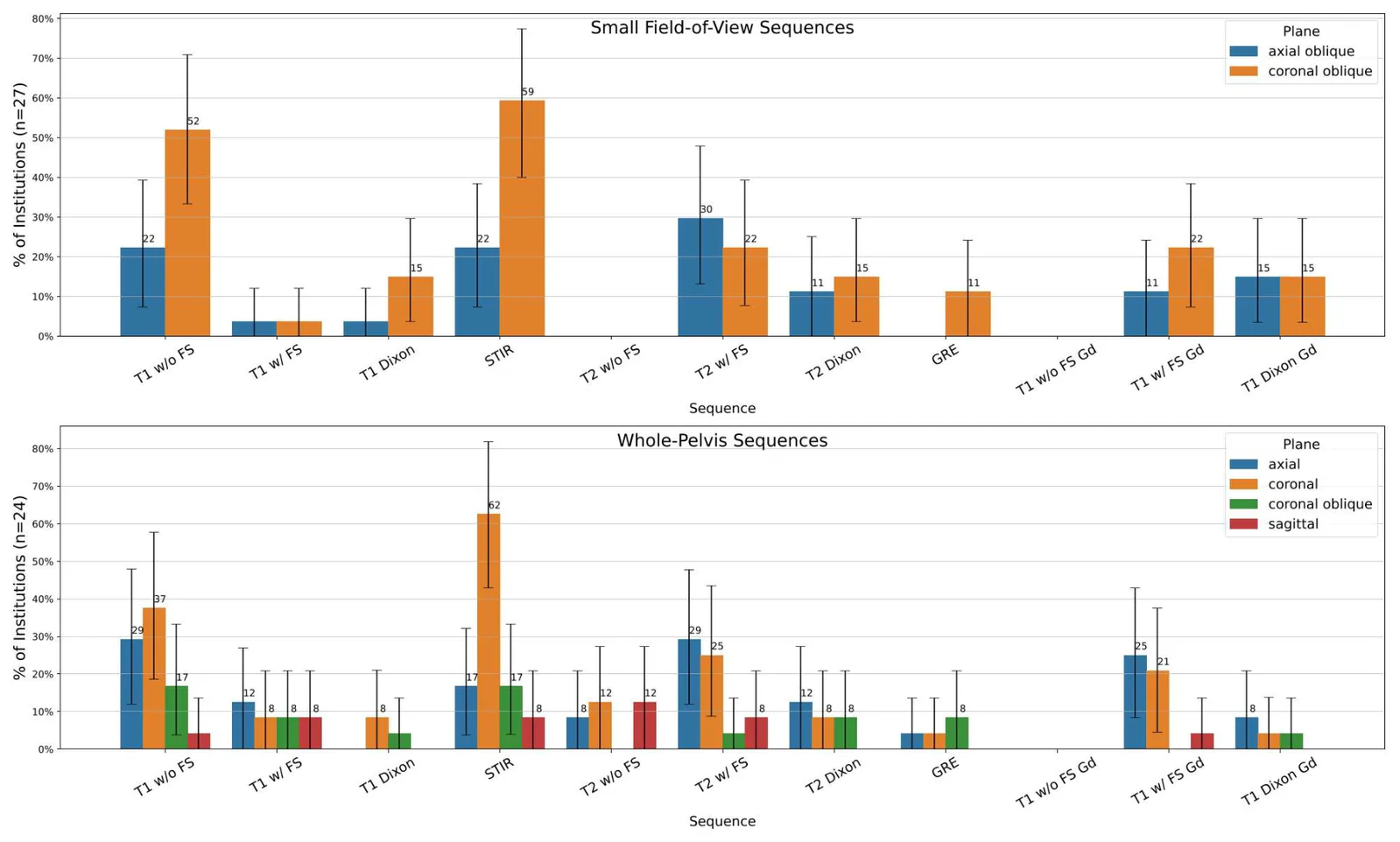
Distribution of the most common sequences and planes being used in SIJ imaging at the institutions of survey respondents. Values represent the proportion of institutions performing each sequence-plane for the small field-of-view and whole-pelvis option sets, respectively, and using one response per each unique institution (*n* = 32). Confidence intervals represent the 95% percentiles of responses from bootstrap resampling of respondents 10,000 times. For a multiple-response-adjusted variant of these results (i.e., where sequences in shorter protocols receive higher weight), see Figure A1. Abbreviations: Dixon, Dixon technique; FS, Fat Suppression; Gd, Gadolinium; GRE, Gradient Echo; STIR, Short Tau Inversion Recovery; T1, T1-weighted; T2, T2-weighted; w/, with; w/o, without.

**Figure A1:**
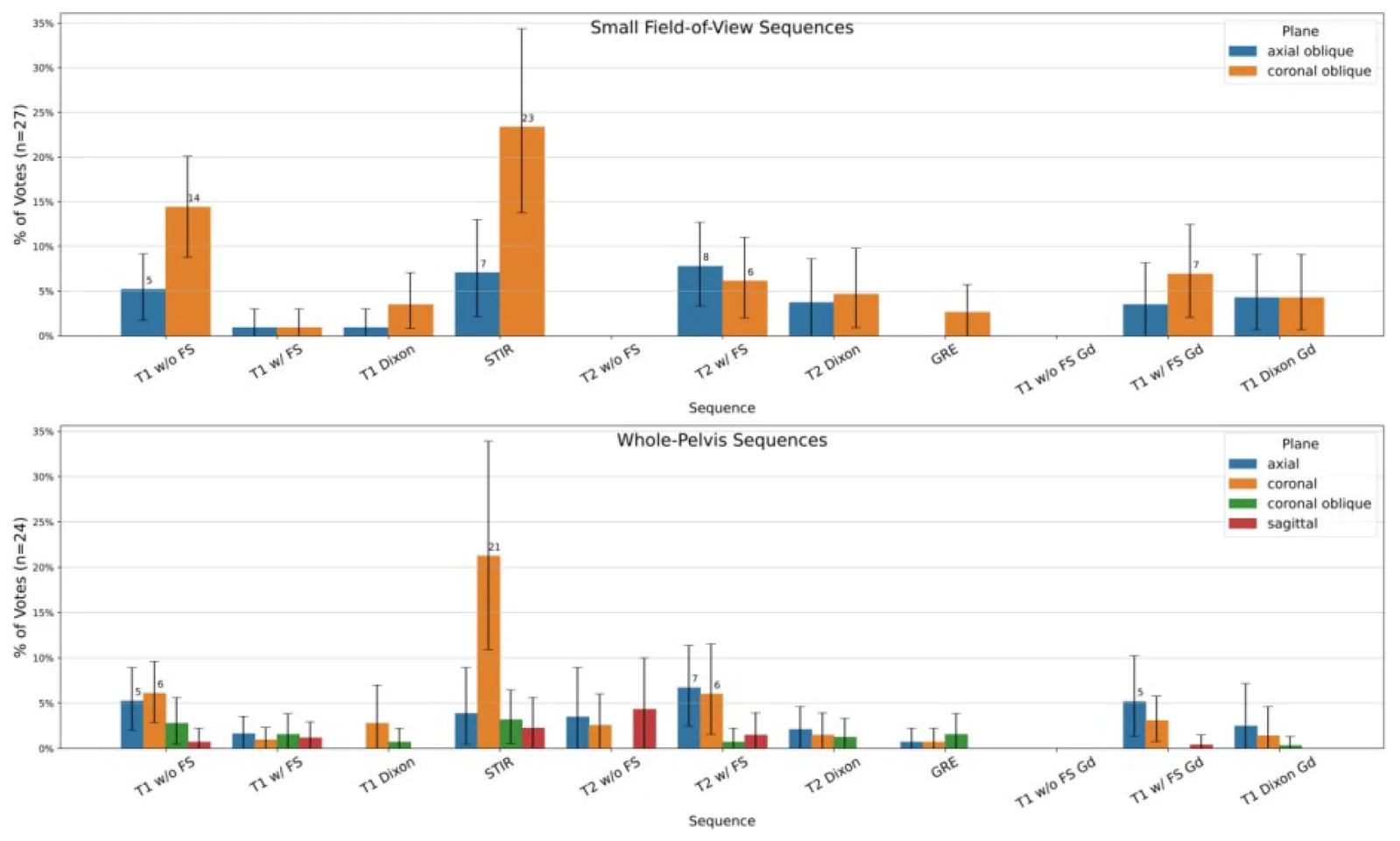
Distribution of the most common sequences and planes being used in SIJ imaging at the institutions of survey respondents, weighted-vote analysis.

In the home institutions of respondents, 75% of centers (*n* = 24 of 32 unique institutions) perform some type of sequence of the entire pelvis as part of their SIJ protocol. Based on both the raw prevalence (Table 2) and the the multiplicity-adjusted prevalence of sequence–plane combination options (Table A1, i.e., inclusions in shorter protocols carrying more weight) the coronal short tau inversion recovery (STIR) was the most common unenhanced whole-pelvis sequence (performed in 62% of the 24 institutions performing whole-pelvis sequences, 21% of votes in all whole-pelvis sequences). The second most commonly performed whole-pelvis sequence was coronal T1-weighted without fat suppression (T1 w/o FS) sequence (37% of institutions, 6% of sequences), followed by axial T1 w/o FS and axial T2-weighted with fat suppression (T2 w/FS) (29% of institutions each, 5% and 7% of sequences, respectively; see Figure 1, Table 2 and Figure A1). The vast majority (93%, *n* = 25 of 27) of institutions selected the posterior cortex of the 2nd sacral vertebral body as the landmark they use to obtain the coronal oblique plane. Of all institutions, a minority (22%, *n* = 7 of 32) utilize GRE sequences.

Most institutions of respondents also perform small field-of-view (FOV) imaging (84%, *n* = 27 of 32 institutions), all of which perform this sequence in the coronal oblique plane, with most also including an axial oblique plane (78%, *n* = 21 of 27). The small FOV sequences for this plane are most often run as a coronal oblique STIR (59% of the 27 institutions, 23% of weighted votes of all small FOV sequences) or coronal oblique T1 w/o FS (52% of institutions or 14% of sequences), with other FS T2W, T2W DIXON and post-contrast sequences being used less commonly (Table 2, Figure 1, and Figure A1). The small FOV axial oblique plane sequences were typically performed as a T2 w/FS or STIR sequence (30% and 22% institutions, or 8% and 7% of all small FOV sequences, respectively), whereas a smaller proportion of sequences were non-FS T1W sequences (22% of institutions, or 5% of sequences).

When asked a free-text question on which sequences they considered essential for SIJ imaging in JSpA, the most cited sequences were small FOV non-FS coronal oblique T1W (52%, *n* = 16 of 31 free-text respondents) and small FOV coronal oblique STIR (42%, *n* = 13 of 31). Furthermore, axial oblique sequences were listed by 35% (*n* = 11 of 31) of respondents and GRE sequences by 19% (*n* = 6 of 31) of respondents.

### 3.3. Use of Intravenous Contrast Material

Most institutions (69%, *n* = 20 of 29) do not routinely administer contrast material as part of the standard SIJ protocol (Table 1). When asked if administration of intravenous contrast was part of their institution’s standard SIJ protocol, participants from 31% of institutions (*n* = 9 of 29) responded ‘yes’, 28% responded ‘no’ and 41% reported that they sometimes use contrast as an ‘optional sequence’. To the opinion question “Is contrast necessary?” 79% of participants (*n* = 27 of 34) said ‘no’, and 21% said ‘yes’. When asked the question “Is contrast helpful?” 57% (*n* = 20 of 35) replied ‘yes’, and 43% replied ‘no’. If they answered ‘yes’ to this question, participants were asked to provide specific findings that would benefit from the use of contrast material in a free-text comment. Participants identified that contrast is helpful to increase confidence in cases with minimal inflammatory disease or equivocal findings on non-contrast sequences. Other comments mentioned that contrast could be helpful in cases where there was concern for infectious sacroiliitis.

### 3.4. Preferred Sequence to Evaluate Specific Findings

**Figure 2:**
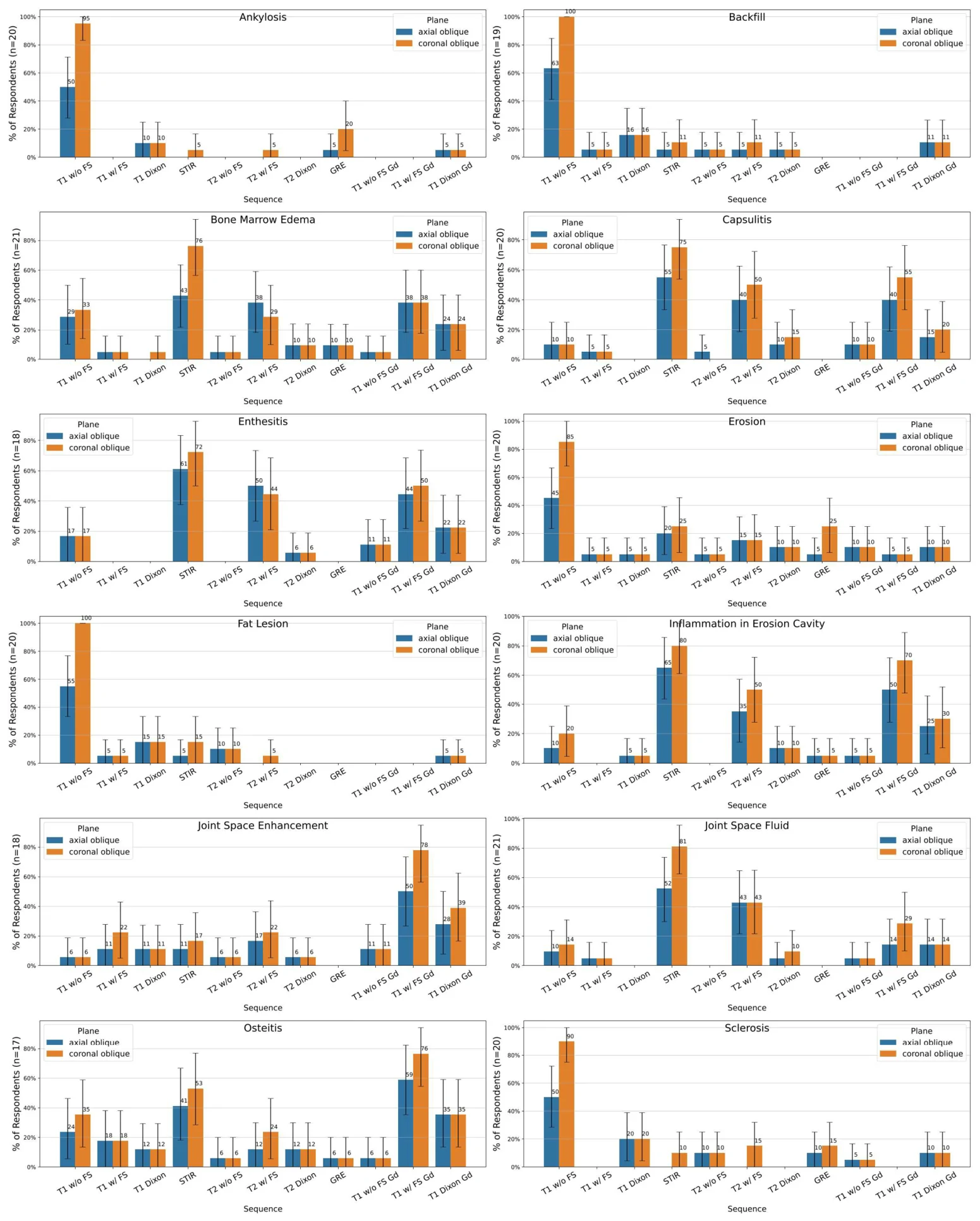
Distribution of respondent preferences on the preferred sequence-plane combinations for various inflammatory and osteochondral damage lesions. Values represent the proportion of respondents who selected each sequence-plane for each of the lesions. Confidence intervals represent the 95% percentiles of responses from bootstrap resampling of respondents 10,000 times. For a multiple-response-adjusted variant of these results (i.e., where sequences in shorter protocols receive higher weight), see Figure A2.

Regarding lesions in the inflammatory domain of the JAMRIS scoring system, the coronal oblique STIR sequence was most commonly selected as a preferred sequence for the evaluation of five of the seven items: bone marrow edema (76% of respondents, 27% of weighted part-votes), capsulitis (75%, 23%), enthesitis (72%, 23%), inflammation in an erosion cavity (80%, 24%), and joint space fluid (81%, 31%; see Table 2, Figure 2, and Table A1). For the remaining two items, lesions which by definition require contrast administration according to the JAMRIS scoring system (i.e., joint space enhancement and osteitis) [11], the small FOV contrast-enhanced coronal oblique T1 w/FS sequence was most commonly selected (78% and 76% of respondents and 35% and 29% of part-votes, respectively).

Concerning the damage domain of the JAMRIS scoring system, the coronal oblique T1 w/o FS sequence was the preferred sequence for evaluation of all five lesions in this category, including sclerosis, erosions, fat lesions, backfill, and ankylosis (85–100% of participants, 36–60% of part-votes) (see Table 2, Figure 2, and Table A1). This was followed by a coronal oblique GRE sequence (14%, CI:3–27%) for evaluation of erosions, and the axial oblique T1 w/o FS for the other lesions. For erosions specifically, 17 respondents selected the coronal oblique T1 w/o FS sequence in their set of preferred sequences, and five selected the GRE, with four selecting both.

## 4. Discussion

The results of this survey of an international group with expertise in interpreting SIJ MRI in pediatric patients with JSpA confirmed the variability of protocols at different institutions. As mentioned in the introduction, the ASAS-SPARTAN international consensus MRI protocol for evaluating adult SIJs has been adopted by some radiology societies. However, while acknowledging the potential application of their recommendations to pediatric imaging, they declined to make specific pediatric recommendations, citing the greater complexities of the interpretation of SIJ MR images in children. These complexities arise from developmental changes that occur in the SIJs throughout childhood at different ages in girls versus boys.

The sacrum is formed from five primary and multiple smaller secondary ossification centers with rudimentary intervertebral disc spaces fusing in the upper sacrum throughout childhood and within the lower sacrum in early adulthood [12,13]. Furthermore, in younger children, open lateral sacral apophyses cause cartilaginous widening at junctional zones between the SIJs and segmental apophyses of the sacrum. It is in these areas where ossified secondary ossification centers eventually form [14,15]. These developmental processes occur earlier and are completed earlier in girls versus boys [14].

These developmental changes lead to increased subchondral signal intensity on fluid-sensitive MR sequences at metaphyseal equivalent regions of the lateral sacral apophysis, which is sometimes termed ‘flaring’ [7,16]. This ‘flaring’ often predominates on the sacral side of the SIJs and is typically symmetric. In addition to signal changes related to cartilage ossification, hematopoietic red marrow predominates in younger children and undergoes conversion to more yellow marrow in older children and adolescents. These signal changes at and adjacent to the SIJs can be confused with inflammatory changes. There are other developmental features in the pediatric SIJ which may be confused with damage changes. The subchondral bone plates of the sacral and iliac bones at the SIJs may appear blurred and irregular in skeletally immature patients in comparison to the low signal intensity black line normally seen in skeletally mature patients. This blurring of the subchondral bone plate may be exacerbated in children on T1-weighted sequences due to the underlying red marrow having intermediate to low signal intensity on this sequence. Unlike the ASAS-SPARTAN international consensus protocol, the ESSR-ESPR European consensus protocol is specific to children. However, the ESSR-ESPR consensus protocol does not include a recommendation for a thin 3D GRE sequence as the ASAS-SPARTAN consensus protocol does, which is likely important given the limitations of evaluating erosions at the pediatric SIJ on T1-weighted sequences. The greater complexities in the interpretation of pediatric SIJ MRI, the lack of an international pediatric-specific protocol, and the variability of protocols used at different institutions queried in this survey highlight the need for an international consensus protocol specific to pediatrics.

In addition to documenting current international practice, this survey also identified areas of general agreement, including the widespread use of the posterior cortex of the 2nd sacral vertebral body to plan the coronal oblique plane. This is in agreement with the recently published ASAS-SPARTAN collaborative adult SIJ protocol [6]. The use of this landmark allows for consistency in the alignment of the coronal oblique plane, widely considered the most important plane when evaluating the SIJs because the landmark is easily identified at all ages and is not subject to anatomical variation [4,6]. Furthermore, small FOV coronal oblique images were most often obtained utilizing non-FS T1W and STIR sequences, and these sequences were commonly selected as the most useful for diagnosis of lesions categorized as part of the damage (coronal oblique T1W) and inflammatory (coronal oblique STIR), respectively. The use of these sequences is also in agreement with the 2024 ASAS-SPARTAN adult (adopted by the European Society of Radiology (ESR) and European Society of Musculoskeletal Radiology [ESSR]) and with the 2020 European ESSR-ESPR recommended pediatric protocols for SIJ evaluation [4,6].

The use of the axial oblique plane, while not universal, was utilized by the majority of respondents, with most performing it as a FS T2W sequence. Both the ASAS-SPARTAN adult and the European ESSR-ESPR recommended pediatric protocols include an axial FS T2W sequence [4,6]. The reasoning for obtaining an axial oblique sequence is in keeping with the general principle in joint imaging of assessment in at least two planes that are orthogonal to the joint and perpendicular to each other [6]. This allows for more precise localization of findings in relation to the appropriate anatomic location. The recommendation to employ a fluid-sensitive sequence is to enable evaluation of inflammatory lesions, particularly bone marrow edema, on two orientations to assist in the identification of false positive findings due to phase encoding artefacts, partial volume averaging that mimics bone marrow edema (BME) or BME related to mechanical stress, which is observed most frequently in the antero-inferior portion of the joint [6]. However, there is not yet a consensus at pediatric institutions with regard to whether the axial plane should be oriented in an oblique plane perpendicular to the coronal oblique plane or in a plane perpendicular to the long axis of the body [11]. Aligning the axial plane in relation to the long axis of the body allows for evaluation of the remainder of the pelvis and hips in a standard axial plane. This is helpful given that hip joint arthritis and pelvic enthesitis are commonly seen concomitantly in children with JSpA on SIJ MRI [10]. Additionally, hip disease is a poor prognostic factor in JSpA and further supports including it in the imaging views. Whether standard axial imaging of the whole pelvis, axial oblique imaging as a small FOV of the SIJs or axial oblique images including the whole pelvis should be preferred in clinical trials of SIJs remains an open question in the pediatric population.

Respondents from a minority of institutions (22%) reported that they utilized Gradient Recalled Echo (GRE) sequences in the assessment of SIJs in JSpA at their centers. The use of GRE sequences has been shown to improve the identification of structural changes at the SIJs in adults. GRE, zero echo time (ZTE), susceptibility weighted MRI (SWI) and synthetic CT sequences have all been shown to be comparable to CT for the bony assessment of the SIJs in adults and superior to T1-weighted sequences [17,18,19]. These and other thin slice 3D-GRE sequences have been referred to as erosion-sensitive sequences when used in SIJ imaging [6]. A recent study by Herregods et al. demonstrated the challenges of using non-FS T1W images to diagnose erosions in children utilizing the current definitions of erosion, which define erosions based on the loss of hypointense T1 cortical bone signal at the osteochondral interface [20]. Using non-FS T1W images, the authors found that approximately 65% of SIJs in children without SIJ disease have at least one feature that would be considered abnormal in adult SIJs [20]. These authors conclude that “T1-weighted images are less suitable for detection of erosions in pediatric SI joints than in adults. A useful pediatric-specific definition of SI joint erosion is likely to require a change in the magnetic resonance imaging acquisition protocol to include an erosion-sensitive sequence in addition to T1-weighted imaging” [20]. We hypothesize that the low reported use of a GRE sequence or other erosion-sensitive sequence in our survey relates to the fact that much of the literature stressing the importance of these sequences in the assessment of pediatric SIJs is recent and has not yet been translated into clinical practice in most pediatric imaging centers [6,18,20]. However, it should be noted that a coronal oblique GRE sequence was listed second to the coronal oblique non-FS T1W sequence in the evaluation of erosions in our survey and was commonly listed as an essential sequence by those who are using this sequence in clinical practice.

Our study showed a discrepancy regarding the lack of utilization of contrast material for assessment of pediatric SIJs, as most of our survey participants responded that they did not routinely administer intravenous contrast material as part of the standard SIJ protocol, but slightly over half believed that contrast can be helpful. This lack of consensus is reflected in the literature. Several studies have questioned the utility of contrast material in the evaluation of sacroiliitis, particularly in patients with established disease [21,22,23]. In a study evaluating contrast versus fluid-sensitive sequences alone in children with JSpA, Weiss et al. found that while contrast did facilitate the detection of synovitis, this finding was generally concomitant with other signs of inflammation (e.g., bone marrow edema or capsulitis) which were captured on fluid-sensitive sequences, leading the authors to conclude that contrast was not necessary to diagnose active inflammation [22]. However, other studies in pediatric patients have reported rates of synovial enhancement in the SIJ without bone marrow edema in approximately 1/3 of the cases [10,24]. Herregods et al. found that a very low percentage of SIJs with synovial enhancement may not show concomitant increased signal on fluid-sensitive sequences, leading these authors to speculate that “contrast-enhanced images may play a limited role in establishing the diagnosis of sacroiliitis” [24]. Other studies have subsequently shown that for calibrated readers, sensitivity and reliability for scoring inflammatory lesions was greater on fluid-sensitive versus contrast-enhanced sequences [3]. The results of our survey concerning the overall lack of utilization of contrast material for assessment of SIJs in the pediatric population aligns with the information from the current literature pointing out that contrast is typically not necessary in the evaluation of SIJs in JSpA for standard clinical protocols, but may be used as an optional sequence when establishing an initial diagnosis with presence of minimal findings or in the setting where other entities (e.g., septic arthritis) are in the differential diagnosis.

There are limitations in this study. First, the survey did not include questions regarding less commonly used sequences such as DWI as pre-set checkbox options; although free-text entry was possible, this may have led to under-reporting of these sequences. Second, there were no questions specifically aimed at capturing the differences in practice or preference between imaging at 1.5 T or 3 T MRI, or between different MRI platforms. Other relevant questions the survey did not capture include the potential differences in sequences between initial diagnosis versus follow-up monitoring, and how readers adjust their interpretation of bone marrow changes according to the patient’s age. Also, the survey did not collect the approximate volume of pediatric patients with JSpA managed or imaged at the participating institutions. These omissions were due to practical limitations on survey length and should be investigated in future studies once the recommended sequence sets are more narrowly defined. Additionally, despite sending the survey to an international group of radiologists and rheumatologists, the majority of respondents were from North America and Europe, with only a small number of respondents from Asia, Australia, and South America. Also, only two rheumatologists responded to the survey, which may reflect that not all rheumatologists interpret MRI exams or have knowledge of specific sequences and planes performed at their institutions. Furthermore, the response rate of our survey was only 43%, which raises the possibility of non-response bias. Given the purposive/convenience recruitment strategy and relatively modest response rate, individuals with a particular interest in pediatric SIJ imaging or MRI protocol standardization may have been more likely to participate. Therefore, the observed practice patterns may not fully represent the broader international community.

## 5. Conclusions

This survey confirmed the variability of SIJ MRI acquisition protocols among a group of international experts in the evaluation of MR findings of sacroiliitis in JSpA. Points of general agreement are in keeping with a previously published international consensus for adults and a European consensus for pediatric SIJ MRI, including the use of the dorsal cortex of the S2 vertebral body to plan coronal oblique sequences, the use of two small FOV coronal oblique sequences (non-FS T1, and STIR or T2 FS), and that the standard clinical protocol does not need post-contrast imaging. Points of disagreement include identifying the best scan plane and FOV for the axial sequence, and whether to incorporate an erosion-sensitive sequence into the standard protocol.

## Figures and Tables

**Table 1:** Survey response summary for general imaging protocol questions. The questions are listed in condensed form. Options are Yes and No, unless indicated otherwise in brackets. Confidence intervals represent the 95% percentiles of responses from bootstrap resampling of respondents 10,000 times. Abbreviations: CI, Confidence Interval; IQR, length of interquartile range; SIJ, Sacroiliac Joint; WBMRI, whole-body magnetic resonance imaging.

	Total Responses	Yes/Option 1		No/Option 2		Option 3	
Options	N	Value	95% CI	Value	95% CI	Value	95% CI
Institutional Practice Questions	N of Institutions						
Do you administer contrast(y/n/optional)?	29	31%	(15–48)	28%	(12–44)	41%	(24–59)
Dorsal cortex of S2 for Landmark?	27	93%	(81–100)	7%	(0–19)		
Dedicated SIJ with WBMRI (y/n/sometimes)?	26	62%	(42–80)	11%	(0–25)	27%	(10–45)
Magnet strength (1.5T/3T/both):	24	12%	(0–28)	13%	(0–27)	75%	(57–92)
Imaging time (median, IQR):	23	29	(25–30)	9	(5–20)		
Imaging demographic (children/adults/both):	25	72%	(52–88)	0%	(0–0)	28%	(12–48)
Different protocols for children and adults?	7	43%	(0–83)	57%	(17–100)		
Respondent Opinion Questions	N of Respondents						
Respondent years of experience (median, IQR)	36	15	(12–18)	9	(5–14)		
Is contrast necessary?	34	21%	(8–35)	79%	(65–92)		
Is contrast helpful?	35	57%	(40–74)	43%	(26–60)		
Pre-contrast ordering preference?	32	56%	(39–73)	44%	(27–61)		
Post-contrast ordering preference?	30	27%	(11–43)	73%	(57–89)		

**Table 2:** Top sequences with the most votes for current imaging practice of SIJ imaging and respondent preference for each JAMRIS-SIJ item. Votes columns represent the proportion of institutions or respondents selecting each sequence-plane combination for each of the JAMRIS-SIJ domain items or protocol type (*n* = 38 respondents from at least 32 institutions). The current practice fields aggregated one response per institution, whereas the item-wise opinion summaries aggregated all respondents. Confidence intervals represent the 95% percentiles of responses from bootstrap resampling of respondents 10,000 times. See Figure 2 for the full set of sequence-plane options. For a multiple-response-adjusted variant of these results (i.e., where sequences in shorter protocols receive higher weight), see Table A1. Abbreviations: Ax, Axial; CI, Confidence Interval; Cor, Coronal; Dixon, Dixon technique; FS, Fat Suppression; Gd, Gadolinium; GRE, Gradient Echo; ob, oblique; STIR, Short Tau Inversion Recovery; T1, T1-weighted; T2, T2-weighted; w/, with; w/o, without;.

	Total Responses	Top Sequence	Votes	95% CI	2nd Most Selected Sequence	Votes	95% CI	3rd Most Selected Sequence	Votes	95% CI	4th Most Selected Sequence	Votes	95% CI
Current Practice	N of Institutions												
Small Field-of-View Sequences	27	Cor ob STIR	59%	(40–77)	Cor ob T1 w/o FS	52%	(33–71)	Ax ob T2 w/ FS	30%	(13–48)	Cor ob T2 w/ FS	22%	(8–39)
Whole-Pelvis Sequences	24	Cor STIR	62%	(43–82)	Cor T1 w/o FS	37%	(19–58)	Ax T1 w/o FS	29%	(12–48)	Ax T2 w/ FS	29%	(12–48)
Respondent Opinions	N of Respondents												
(1) Inflammatory Domain													
Bone Marrow Edema	21	Cor ob STIR	76%	(57–94)	Ax ob STIR	43%	(22–64)	Ax ob T1 w/ FS Gd	38%	(18–60)	Cor ob T1 w/ FS Gd	38%	(18–60)
Capsulitis	20	Cor ob STIR	75%	(54–94)	Cor ob T1 w/ FS Gd	55%	(33–76)	Ax ob STIR	55%	(33–76)	Cor ob T2 w/ FS	50%	(28–72)
Enthesitis	18	Cor ob STIR	72%	(50–93)	Ax ob STIR	61%	(38–83)	Ax ob T2 w/ FS	50%	(27–73)	Cor ob T1 w/ FS Gd	50%	(27–74)
Inflammation in Erosion Cavity	20	Cor ob STIR	80%	(61–95)	Cor ob T1 w/ FS Gd	70%	(48–89)	Ax ob STIR	65%	(43–86)	Ax ob T1 w/ FS Gd	50%	(28–72)
Joint Space Enhancement	18	Cor ob T1 w/ FS Gd	78%	(56–95)	Ax ob T1 w/ FS Gd	50%	(27–73)	Cor ob T1 Dixon Gd	39%	(17–62)	Ax ob T1 Dixon Gd	28%	(8–50)
Joint Space Fluid	21	Cor ob STIR	81%	(62–96)	Ax ob STIR	52%	(30–74)	Ax ob T2 w/ FS	43%	(22–65)	Cor ob T2 w/ FS	43%	(22–65)
Osteitis	17	Cor ob T1 w/ FS Gd	76%	(55–94)	Ax ob T1 w/ FS Gd	59%	(35–82)	Cor ob STIR	53%	(29–77)	Ax ob STIR	41%	(18–67)
(2) Damage Domain													
Ankylosis	20	Cor ob T1 w/o FS	95%	(83–100)	Ax ob T1 w/o FS	50%	(28–71)	Cor ob GRE	20%	(5–40)	Ax ob T1 Dixon	10%	(0–25)
Backfill	19	Cor ob T1 w/o FS	100%	(100–100)	Ax ob T1 w/o FS	63%	(41–85)	Ax ob T1 Dixon	16%	(0–35)	Cor ob T1 Dixon	16%	(0–35)
Erosion	20	Cor ob T1 w/o FS	85%	(68–100)	Ax ob T1 w/o FS	45%	(24–67)	Cor ob STIR	25%	(6–45)	Cor ob GRE	25%	(6–45)
Fat Lesion	20	Cor ob T1 w/o FS	100%	(100–100)	Ax ob T1 w/o FS	55%	(33–77)	Ax ob T1 Dixon	15%	(0–33)	Cor ob T1 Dixon	15%	(0–33)
Sclerosis	20	Cor ob T1 w/o FS	90%	(75–100)	Ax ob T1 w/o FS	50%	(29–72)	Ax ob T1 Dixon	20%	(5–39)	Cor ob T1 Dixon	20%	(5–39)

**Figure A2:**
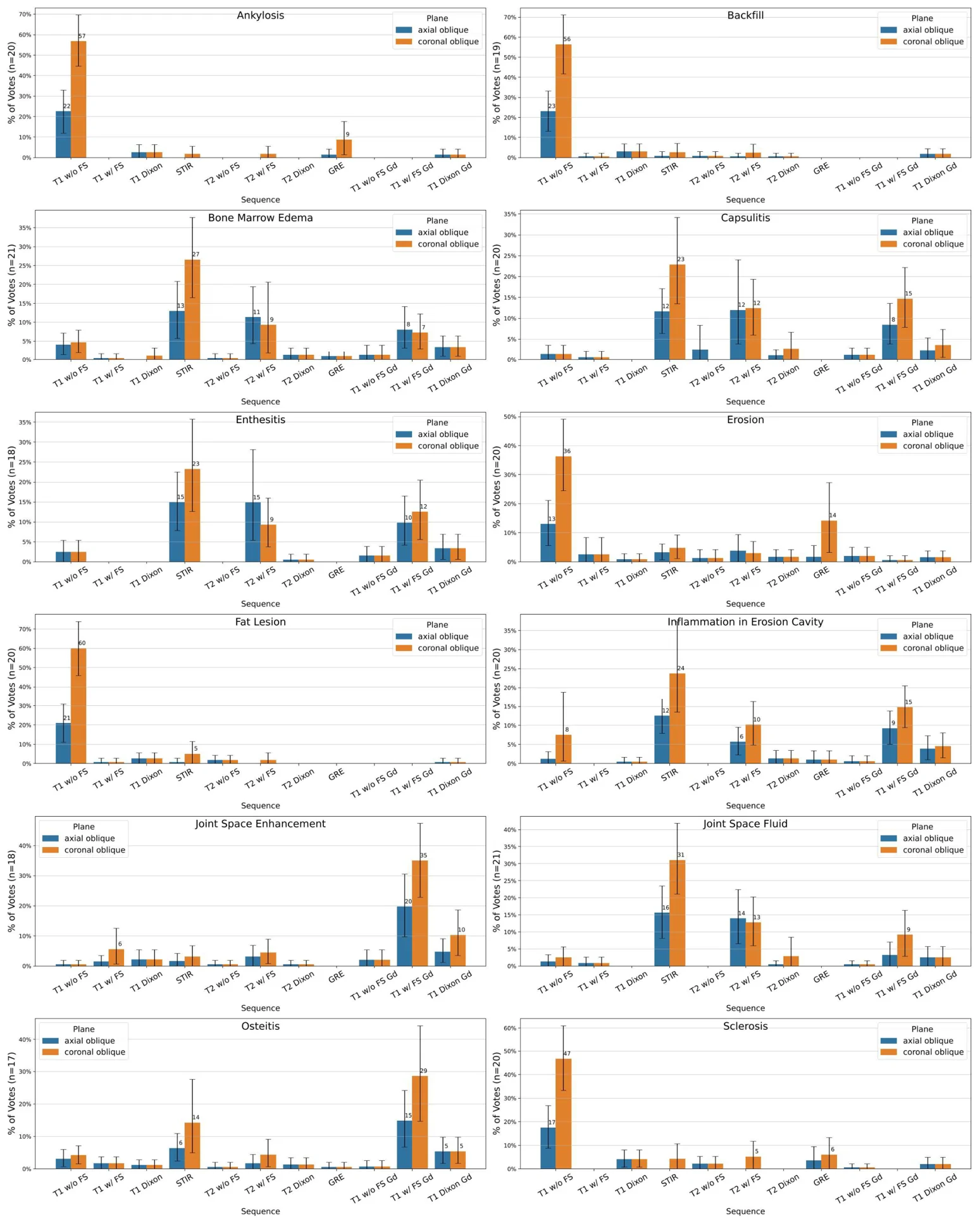
Distribution of respondent preferences on the preferred sequence-plane combinations for various inflammatory and osteochondral damage lesions.

## Data Availability

The data that support the findings of this study are available from the authors upon request.

## Supplementary Materials

The following supporting information can be downloaded at: https://www.mdpi.com/article/10.3390/jcm15176816/s1. Supplementary File S1: Complete survey instrument used in the study.

## Abbreviations

The following abbreviations are used in this manuscript:

SIJs	Sacroiliac Joints
JIA	Juvenile Idiopathic Arthritis
ASpA	Adult Spondylarthritis
JSpA	Juvenile Spondylarthritis
OMERACT	Outcomes Measures in Rheumatology and Clinical Trials
JAMRIS	Juvenile Idiopathic Arthritis MRI SIJ working group
ESSR	European Society of Musculoskeletal Radiology
ESPR	European Society of Paediatric Radiology
MRI	Magnetic Resonance Imaging
CT	Computed Tomography
DWI	Diffusion-Weighted Imaging
GRE	Gradient Echo
ZTE	Zero Echo Time
SWI	Susceptibility-Weighted MRI
FOV	Field of View
STIR	Short Tau Inversion Recovery
BME	Bone Marrow Edema
ASAS	SpondyloArthritis International Society
SPARTAN	Spondyloarthritis Research and Treatment Network

## Appendix A

Top sequences with the most votes for current imaging practice of SIJ imaging and respondent preference for each JAMRIS-SIJ item. Vote column represents the total multiple-response-adjusted proportion of part-votes given to the respective sequence-plane combinations for each of the JAMRIS-SIJ domain items or protocol type at the respondents’ institutions (n = 38 respondents from at least 32 institutions). Plane-wise distribution of votes is the sum of part-votes given to all sequences in in these planes, where (-) indicates planes not represented in small field-of-view question options. The current practice fields aggregated one response per institution, whereas the item-wise opinion summaries aggregated all respondents. Confidence intervals represent the 95% percentiles of responses from bootstrap resampling of respondents 10,000 times. See Figure A2 for the full set of sequence-plane options. Abbreviations: Ax, Axial; CI, Confidence Interval; Cor, Coronal; Dixon, Dixon technique; FS, Fat Suppression; Gd, Gadolinium; GRE, Gradient Echo; ob, oblique; Sag, Sagittal; STIR, Short Tau Inversion Recovery; T1, T1-weighted; T2, T2-weighted; w/, with; w/o, without; -, not applicable.

**Table A1:** Top sequences with the most votes for current imaging practice of SIJ imaging and respondent preference for each JAMRIS-SIJ item.

	Total Responses	Axial (ob)/ Cor /Cor ob/Sag Proportions of Vote (%)	Total % of Top 4 Sequences	Top Sequence	Votes	95% CI	2nd Most Selected Sequence	Votes	95% CI	3rd Most Selected Sequence	Votes	95% CI	4th Most Selected Sequence	Votes	95% CI
Current Practice	N of Institutions														
Small Field–of–View Sequences	27	33/–/67/–	53%	Cor ob STIR	23%	(14–34)	Cor ob T1 w/o FS	14%	(9–20)	Ax ob T2 w/ FS	8%	(3–13)	Ax ob STIR	7%	(2–13)
Whole–Pelvis Sequences	24	31/46/12/10	40%	Cor STIR	21%	(11–34)	Ax T2 w/ FS	7%	(3–11)	Cor T1 w/o FS	6%	(3–10)	Cor T2 w/ FS	6%	(2–12)
Respondent Opinions	N of Respondents														
1) Inflammatory Domain															
Bone Marrow Edema	21	44/–/56/–	60%	Cor ob STIR	27%	(17–38)	Ax ob STIR	13%	(6–21)	Ax ob T2 w/ FS	11%	(4–19)	Cor ob T2 w/ FS	9%	(2–20)
Capsulitis	20	41/–/59/–	62%	Cor ob STIR	23%	(13–34)	Cor ob T1 w/ FS Gd	15%	(8–22)	Cor ob T2 w/ FS	12%	(6–19)	Ax ob T2 w/ FS	12%	(4–24)
Enthesitis	18	47/–/53/–	65%	Cor ob STIR	23%	(13–36)	Ax ob T2 w/ FS	15%	(5–28)	Ax ob STIR	15%	(8–23)	Cor ob T1 w/ FS Gd	12%	(6–20)
Inflammation in Erosion Cavity	20	36/–/64/–	61%	Cor ob STIR	24%	(13–37)	Cor ob T1 w/ FS Gd	15%	(9–21)	Ax ob STIR	12%	(8–17)	Cor ob T2 w/ FS	10%	(5–16)
Joint Space Enhancement	18	36/–/64/–	70%	Cor ob T1 w/ FS Gd	35%	(23–47)	Ax ob T1 w/ FS Gd	20%	(9–30)	Cor ob T1 Dixon Gd	10%	(3–19)	Cor ob T1 w/ FS	6%	(1–13)
Joint Space Fluid	21	38/–/62/–	73%	Cor ob STIR	31%	(21–42)	Ax ob STIR	16%	(8–24)	Ax ob T2 w/ FS	14%	(6–22)	Cor ob T2 w/ FS	13%	(6–20)
Osteitis	17	38/–/62/–	64%	Cor ob T1 w/ FS Gd	29%	(14–44)	Ax ob T1 w/ FS Gd	15%	(7–24)	Cor ob STIR	14%	(5–28)	Ax ob STIR	6%	(3–11)
2) Damage Domain															
Ankylosis	20	28/–/72/–	90%	Cor ob T1 w/o FS	57%	(44–69)	Ax ob T1 w/o FS	22%	(13–33)	Cor ob GRE	9%	(1–18)	--	<5%	--
Backfill	19	32/–/68/–	86%	Cor ob T1 w/o FS	56%	(41–71)	Ax ob T1 w/o FS	23%	(13–33)	--	<5%	--	--	<5%	--
Erosion	20	32/–/68/–	68%	Cor ob T1 w/o FS	36%	(24–49)	Cor ob GRE	14%	(3–27)	Ax ob T1 w/o FS	13%	(6–21)	Cor ob STIR	5%	(1–9)
Fat Lesion	20	28/–/72/–	88%	Cor ob T1 w/o FS	60%	(46–74)	Ax ob T1 w/o FS	21%	(11–31)	Cor ob STIR	5%	(0–11)	--	<5%	--
Sclerosis	20	30/–/70/–	75%	Cor ob T1 w/o FS	47%	(33–61)	Ax ob T1 w/o FS	17%	(9–27)	Cor ob GRE	6%	(0–13)	Cor ob T2 w/ FS	5%	(0–12)

Distribution of the most common sequences and planes being used in SIJ imaging at the institutions of survey respondents, weighted-vote analysis. Values represent the proportion of total (part-)votes each sequence-plane received within the small field-of-view and whole-pelvis option sets, respectively, weighing multiple selections equally per respondent, and using one response per each unique institution (n = 32). Confidence intervals represent the 95% percentiles of responses from bootstrap resampling of respondents 10,000 times. Abbreviations: Dixon, Dixon technique; FS, Fat Suppression; Gd, Gadolinium; GRE, Gradient Echo; STIR, Short Tau Inversion Recovery; T1, T1-weighted; T2, T2-weighted; w/, with; w/o, without.

Distribution of respondent preferences on the preferred sequence-plane combinations for various inflammatory and osteochondral damage lesions. Values represent the proportion of total votes each sequence-plane received for each of the lesions, weighing multiple selections equally per respondent. Confidence intervals represent the 95% percentiles of responses from bootstrap resampling of respondents 10,000 times. Abbreviations: Dixon, Dixon technique; FS, Fat Suppression; Gd, Gadolinium; GRE, Gradient Echo; STIR, Short Tau Inversion Recovery; T1, T1-weighted; T2, T2-weighted; w/, with; w/o, without.
