# Supplementary material for: International Imaging Practices for Sacroiliac Joint MRI Acquisition Protocols in Patients with Juvenile Spondylarthritis
Source: J Clin Med. 2026 Sep 2;15(17):6816. doi: 10.3390/jcm15176816 (PMC13566237; doi:10.3390/jcm15176816)
Supplement: Supplementary file 1 [file jcm-15-06816-s001.zip › Supplementary File S1.pdf]

**International OMERACT Collaboration towards the Development and Validation of Sacroiliac Joint MRI Scoring System for the Assessment of Juvenile Idiopathic Arthritis Imaging Protocols Survey**

Please enter the name of your institution.

________________________________________

Please indicate your specialty (radiologist, rheumatologist, other clinician, etc.):

________________________________________

How many years of experiece do you have evaluating SIJ MRI?

________________________________

Abbreviations used in this survey:

BME - Bone Marrow Edema

FOV - Field of View

FS - Fat Suppression

GRE - Gradient Echo

JIA - Juvenile Idiopathic Arthritis

SIJ - Sacroiliac Joint

w/ - With

w/o - Without

**Part 1: Please select the MRI sequences that you use at your institution for imaging the sacroiliac joints.**

Pre-contrast ◯ No ◯ Yes

Pre-contrast whole pelvis sequences ◯ No ◯ Yes

| | T1 w/o FS | T1 w/ FS | T1 Dixon | STIR | T2 w/o FS | T2 w/ FS | T2 Dixon |
|---|---|---|---|---|---|---|---|
| Coronal | ☐ | ☐ | ☐ | ☐ | ☐ | ☐ | ☐ |
| Coronal oblique | ☐ | ☐ | ☐ | ☐ | ☐ | ☐ | ☐ |
| Axial | ☐ | ☐ | ☐ | ☐ | ☐ | ☐ | ☐ |
| Sagittal | ☐ | ☐ | ☐ | ☐ | ☐ | ☐ | ☐ |

Pre-contrast small FOV of SIJ ◯ No ◯ Yes

| | T1 w/o FS | T1 w/ FS | T1 Dixon | STIR | T2 w/o FS | T2 w/ FS | T2 Dixon | GRE |
|---|---|---|---|---|---|---|---|---|
| Coronal oblique | ☐ | ☐ | ☐ | ☐ | ☐ | ☐ | ☐ | ☐ |
| Axial oblique | ☐ | ☐ | ☐ | ☐ | ☐ | ☐ | ☐ | ☐ |

Any other pre-contrast sequence(s)? Please specify FOV (whole pelvis or small FOV of SIJs), plane and sequence. ________________________

Post-contrast ◯ No ◯ Yes

Post-contrast whole pelvis sequences ◯ No ◯ Yes

| | T1 w/o FS | T1 w/ FS | T1 Dixon |
|---|---|---|---|
| Coronal | ☐ | ☐ | ☐ |
| Coronal oblique | ☐ | ☐ | ☐ |
| Axial | ☐ | ☐ | ☐ |
| Sagittal | ☐ | ☐ | ☐ |

Post-contrast small FOV of SIJ ◯ No ◯ Yes

| | T1 w/o FS | T1 w/ FS | T1 Dixon |
|---|---|---|---|
| Coronal oblique | ☐ | ☐ | ☐ |
| Axial oblique | ☐ | ☐ | ☐ |

Any other post-contrast sequence(s)? Please specify FOV (whole pelvis or small FOV of SIJs), plane and sequence. ________________________

## Part 2: Miscellaneous SIJ protocol questions

| | | |
|---|---|---|
| 1 | Do you administer intravenous contrast for SIJ MRI at your institution? | ◯ Yes (part of our standard protocol)<br>◯ No (not part of standard protocol)<br>◯ Sometimes (optional sequence) |
| 1a | Is contrast necessary? | ◯ No ◯ Yes |
| 1b | Is contrast helpful? | ◯ No ◯ Yes |
| 1c | If so, please list the specific imaging findings that could benefit from the utilization of contrast in SIJ imaging: | ________________________________ |
| 2 | If you do coronal oblique images to the SIJs, what landmark do you use to angle the coronal plane? | ☐ Dorsal cortex of S2 ☐ Other |
| 2a | If other, please specify the landmark | ________________________________ |

List of sequences you have selected as your institution's SIJ protocol on the previous page (sequences you did not select are followed by a blank ____):

Pre-contrast sequences:
Whole pelvis - Coronal: [pre_wp_coronal]
Whole pelvis - Coronal oblique: [pre_wp_oblique_coronal]
Whole pelvis - Axial: [pre_wp_axial]
Whole pelvis - Sagittal: [pre_wp_sagittal]
Small FOV - Coronal oblique: [pre_s_oblique_coronal]
Small FOV - Axial oblique: [pre_s_oblique_axial]
Any other: [pre_any_other]

Post-contrast sequences:
Whole pelvis - Coronal: [post_wp_coronal]
Whole pelvis - Coronal oblique: [post_wp_oblique_coronal]
Whole pelvis - Axial: [post_wp_axial]
Whole pelvis - Sagittal: [post_wp_sagittal]
Small FOV - Coronal oblique: [post_s_oblique_coronal]
Small FOV - Axial oblique: [post_s_oblique_axial]
Any other: [post_any_other]

| | | |
|---|---|---|
| 3 | Which sequences do you consider essential for imaging the SIJ in JIA?<br><br>Please copy/paste sequences from your institution's list (above) or add missing ones, describing by FOV, plane, sequence, and with/without contrast. | ________________________________ |
| 4 | Which sequences do you consider optional for imaging the SIJ in JIA?<br><br>Please copy/paste sequences from your institution's list (above). | ________________________________ |
| 5 | Are there certain sequences that should be performed first on pre-contrast images to avoid motion artifact?<br><br>(considering, for example, the most important sequences to provide clinically meaningful information should be performed first, etc.) | ◯ No ◯ Yes |

| | | |
|---|---|---|
| 5a | If so, what should be the pre-contrast sequence order? | ____________________ |
| 6 | Are there certain sequences that should be performed first on post-contrast images?<br><br>(considering, for example, the need to finish acquiring the most important sequences that benefit from contrast enhancement before the contrast material extravasates out of the region of interest, etc.) | ◯ No ◯ Yes |
| 6a | If so, what should be the post-contrast sequence order? | ____________________ |
| 7 | If you do whole body MRI for JIA at your institution, do you do dedicated SIJ sequence(s)? | ◯ No ◯ Yes ◯ Not Applicable |
| 7a | If so, which sequence(s)? (plane, sequence, without or with contrast) | ____________________ |
| 8 | Magnet strength: | ◯ 1.5T ◯ 3T ◯ Both 1.5T and 3T |
| 9 | Coil type: | ____________________ |
| 10 | Approximate total scan time: | ____________________ |
| 11 | Imaging demographic: | ◯ Children ◯ Adults<br>◯ Both children and adults |
| 11a | If you image both children and adults, do you have different protocols for children and adults? | ◯ No ◯ Yes |
| 11b | If yes, at what age (or other criteria) do you change to the adult protocol? | ____________________ |

**Part 3: Which sequence(s) are best suited for evaluating the following (A.#) active and (B.#) structural lesions?**

**Please select only the best sequence(s); you do not need to select a sequence from each plane.**

## A.1 Bone Marrow Edema (BME)

View the JAMRIS-SIJ definitions for Inflammatory lesions. ◯ Hide ◯ View

Reference:

Herregods et al., Seminars in Arthritis and Rheumatism, 51 (2021) 1089-1098

**Table 1**
Scoring system for assessing inflammation of thesacroiliac joint by MRI according to the JAMRIS-SIJ scoring system proposed by the MRI in JIA working group of OMERACT. (current version, adapted and updated from reference [11]) (JAMRIS= Juvenile Idiopathic Arthritis MRI Score; SIJ= sacroiliac joint, MRI= Magnetic Resonance Imaging, JIA= Juvenile Idiopathic Arthritis, OMERACT= Outcome Measures in Rheumatology and Clinical Trials; BME= bone marrow edema, FS= fat suppressed).

| Item | Definition | Segmentation/slice | Score range/slice |
|---|---|---|---|
| Bone Marrow Edema (BME) | An ill defined area of high bone marrow signal intensity within the subchondral bone in the ilium or sacrum on fluid sensitive images | 4 quadrants/SIJ | 0–8 |
| BME Intensity | Presence of hyperintensity of the marrow on fluid sensitive images using the signal of the presacral veins or cerebrospinal fluid (CSF) as reference | 1 score /SIJ | 0–2 |
| BME Depth | Continuing increased signal on fluid sensitive images of depth ≥ 5mm/ ≥ 1cm from the articular surface | 1 score /SIJ | 0–2 |
| Osteitis | An ill-defined area of high bone marrow signal intensity within the subchondral bone in the ilium or sacrum on contrast enhanced T1 weighted sequences | 4 quadrants/SIJ | 0–8 |
| Joint Space Fluid | High signal intensity equivalent to the CSF on fluid sensitive sequences within the joint space of the cartilaginous portion of the SIJ | halves/SIJ | 0–4 |
| Joint Space Enhancement | Increased signal intensity on contrast enhanced T1 weighted sequences within the joint space of the cartilaginous portion of the SIJ | halves/SIJ | 0–4 |
| Inflammation in erosion cavity | Increased signal intensity on fluid sensitive or contrast enhanced T1 weighted sequences in an erosion cavity of the cartilaginous portion of the SIJ | halves/SIJ | 0–4 |
| Enthesitis | Increased signal intensity in bone marrow and/or adjacent soft tissue on fluid sensitive or contrast enhanced T1 weighted sequences at sites where ligaments and tendons attach to a bone excluding retroarticular enthesitis | score per case | 0–1 |
| Capsulitis | Increased signal on fluid sensitive or contrast enhanced T1 weighted sequences involving the superior portion of the SIJ capsule | superior halves/SIJ | 0–2 |

Statement of Overarching consideration for all definition: "in comparison to physiological changes normally seen in MRIs of age and sex matched children, and visible in two planes where available"

Pre-contrast sequences, small FOV

| | T1 w/o FS | T1 w/ FS | T1 Dixon | STIR | T2 w/o FS | T2 w/ FS | T2 Dixon | GRE |
|---|---|---|---|---|---|---|---|---|
| Coronal oblique | ☐ | ☐ | ☐ | ☐ | ☐ | ☐ | ☐ | ☐ |
| Axial oblique | ☐ | ☐ | ☐ | ☐ | ☐ | ☐ | ☐ | ☐ |

Other pre-contrast sequence(s):
Please specify FOV (whole pelvis or small FOV of SIJs), plane and sequence ____________________

Post contrast sequences, small FOV

| | T1 w/o FS | T1 w/ FS | T1 Dixon |
|---|---|---|---|
| Coronal oblique | ☐ | ☐ | ☐ |
| Axial oblique | ☐ | ☐ | ☐ |

Other post-contrast sequence(s):
Please specify FOV (whole pelvis or small FOV of SIJs), plane and sequence ____________________

| | |
|---|---|
| Best sequence(s) for BME intensity:<br>Please specify the best sequence(s) from above for evaluating BME intensity, by listing the FOV (whole pelvis or small FOV of SIJs), plane and sequence | ______________________ |
| Best sequence(s) for BME depth:<br>Please specify the best sequence(s) from above for evaluating BME depth, by listing the FOV (whole pelvis or small FOV of SIJs), plane and sequence | ______________________ |

## Part 3: Which sequence(s) are best suited for evaluating the following (A.#) active and (B.#) structural lesions?

**Please select only the best sequence(s); you do not need to select a sequence from each plane.**

### A.2 Osteitis

View the JAMRIS-SIJ definitions for Inflammatory lesions. ○ Hide ○ View

Reference:

Herregods et al., Seminars in Arthritis and Rheumatism, 51 (2021) 1089-1098

**Table 1**
Scoring system for assessing inflammation of thesacroiliac joint by MRI according to the JAMRIS-SIJ scoring system proposed by the MRI in JIA working group of OMERACT. (current version, adapted and updated from reference [11]) (JAMRIS= Juvenile Idiopathic Arthritis MRI Score; SIJ= sacroiliac joint, MRI= Magnetic Resonance Imaging, JIA= Juvenile Idiopathic Arthritis, OMERACT= Outcome Measures in Rheumatology and Clinical Trials; BME= bone marrow edema, FS= fat suppressed).

| Item | Definition | Segmentation/slice | Score range/slice |
|---|---|---|---|
| Bone Marrow Edema (BME) | An ill defined area of high bone marrow signal intensity within the subchondral bone in the ilium or sacrum on fluid sensitive images | 4 quadrants/SIJ | 0–8 |
| BME Intensity | Presence of hyperintensity of the marrow on fluid sensitive images using the signal of the presacral veins or cerebrospinal fluid (CSF) as reference | 1 score /SIJ | 0–2 |
| BME Depth | Continuing increased signal on fluid sensitive images of depth ≥ 5mm/ ≥ 1cm from the articular surface | 1 score /SIJ | 0–2 |
| Osteitis | An ill-defined area of high bone marrow signal intensity within the subchondral bone in the ilium or sacrum on contrast enhanced T1 weighted sequences | 4 quadrants/SIJ | 0–8 |
| Joint Space Fluid | High signal intensity equivalent to the CSF on fluid sensitive sequences within the joint space of the cartilaginous portion of the SIJ | halves/SIJ | 0–4 |
| Joint Space Enhancement | Increased signal intensity on contrast enhanced T1 weighted sequences within the joint space of the cartilaginous portion of the SIJ | halves/SIJ | 0–4 |
| Inflammation in erosion cavity | Increased signal intensity on fluid sensitive or contrast enhanced T1 weighted sequences in an erosion cavity of the cartilaginous portion of the SIJ | halves/SIJ | 0–4 |
| Enthesitis | Increased signal intensity in bone marrow and/or adjacent soft tissue on fluid sensitive or contrast enhanced T1 weighted sequences at sites where ligaments and tendons attach to a bone excluding retroarticular enthesitis | score per case | 0–1 |
| Capsulitis | Increased signal on fluid sensitive or contrast enhanced T1 weighted sequences involving the superior portion of the SIJ capsule | superior halves/SIJ | 0–2 |

Statement of Overarching consideration for all definition: “in comparison to physiological changes normally seen in MRIs of age and sex matched children, and visible in two planes where available”

Pre-contrast sequences, small FOV

| | T1 w/o FS | T1 w/ FS | T1 Dixon | STIR | T2 w/o FS | T2 w/ FS | T2 Dixon | GRE |
|---|---|---|---|---|---|---|---|---|
| Coronal oblique | ☐ | ☐ | ☐ | ☐ | ☐ | ☐ | ☐ | ☐ |
| Axial oblique | ☐ | ☐ | ☐ | ☐ | ☐ | ☐ | ☐ | ☐ |

Other pre-contrast sequence(s):
Please specify FOV (whole pelvis or small FOV of SIJs), plane and sequence ________________

Post-contrast sequences, small FOV

| | T1 w/o FS | T1 w/ FS | T1 Dixon |
|---|---|---|---|
| Coronal oblique | ☐ | ☐ | ☐ |
| Axial oblique | ☐ | ☐ | ☐ |

Other post-contrast sequence(s):
Please specify FOV (whole pelvis or small FOV of SIJs), plane and sequence ________________

## Part 3: Which sequence(s) are best suited for evaluating the following (A.#) active and (B.#) structural lesions?

**Please select only the best sequence(s); you do not need to select a sequence from each plane.**

### A.3 Joint space fluid

View the JAMRIS-SIJ definitions for Inflammatory lesions. ◯ Hide ◯ View

Reference:

Herregods et al., Seminars in Arthritis and Rheumatism, 51 (2021) 1089-1098

**Table 1**
Scoring system for assessing inflammation of thesacroiliac joint by MRI according to the JAMRIS-SIJ scoring system proposed by the MRI in JIA working group of OMERACT. (current version, adapted and updated from reference [11]) (JAMRIS= Juvenile Idiopathic Arthritis MRI Score; SIJ= sacroiliac joint, MRI= Magnetic Resonance Imaging, JIA= Juvenile Idiopathic Arthritis, OMERACT= Outcome Measures in Rheumatology and Clinical Trials; BME= bone marrow edema, FS= fat suppressed).

| Item | Definition | Segmentation/slice | Score range/slice |
|---|---|---|---|
| Bone Marrow Edema (BME) | An ill defined area of high bone marrow signal intensity within the subchondral bone in the ilium or sacrum on fluid sensitive images | 4 quadrants/SIJ | 0–8 |
| BME Intensity | Presence of hyperintensity of the marrow on fluid sensitive images using the signal of the presacral veins or cerebrospinal fluid (CSF) as reference | 1 score /SIJ | 0–2 |
| BME Depth | Continuing increased signal on fluid sensitive images of depth ≥ 5mm/ ≥ 1cm from the articular surface | 1 score /SIJ | 0–2 |
| Osteitis | An ill-defined area of high bone marrow signal intensity within the subchondral bone in the ilium or sacrum on contrast enhanced T1 weighted sequences | 4 quadrants/SIJ | 0–8 |
| Joint Space Fluid | High signal intensity equivalent to the CSF on fluid sensitive sequences within the joint space of the cartilaginous portion of the SIJ | halves/SIJ | 0–4 |
| Joint Space Enhancement | Increased signal intensity on contrast enhanced T1 weighted sequences within the joint space of the cartilaginous portion of the SIJ | halves/SIJ | 0–4 |
| Inflammation in erosion cavity | Increased signal intensity on fluid sensitive or contrast enhanced T1 weighted sequences in an erosion cavity of the cartilaginous portion of the SIJ | halves/SIJ | 0–4 |
| Enthesitis | Increased signal intensity in bone marrow and/or adjacent soft tissue on fluid sensitive or contrast enhanced T1 weighted sequences at sites where ligaments and tendons attach to a bone excluding retroarticular enthesitis | score per case | 0–1 |
| Capsulitis | Increased signal on fluid sensitive or contrast enhanced T1 weighted sequences involving the superior portion of the SIJ capsule | superior halves/SIJ | 0–2 |

Statement of Overarching consideration for all definition: “in comparison to physiological changes normally seen in MRIs of age and sex matched children, and visible in two planes where available”

Pre-contrast sequences, small FOV

| | T1 w/o FS | T1 w/ FS | T1 Dixon | STIR | T2 w/o FS | T2 w/ FS | T2 Dixon | GRE |
|---|---|---|---|---|---|---|---|---|
| Coronal oblique | ☐ | ☐ | ☐ | ☐ | ☐ | ☐ | ☐ | ☐ |
| Axial oblique | ☐ | ☐ | ☐ | ☐ | ☐ | ☐ | ☐ | ☐ |

Other pre-contrast sequence(s):
Please specify FOV (whole pelvis or small FOV of SIJs), plane and sequence ________________

Post-contrast sequences, small FOV

| | T1 w/o FS | T1 w/ FS | T1 Dixon |
|---|---|---|---|
| Coronal oblique | ☐ | ☐ | ☐ |
| Axial oblique | ☐ | ☐ | ☐ |

Other post-contrast sequence(s):
Please specify FOV (whole pelvis or small FOV of SIJs), plane and sequence ________________

## Part 3: Which sequence(s) are best suited for evaluating the following (A.#) active and (B.#) structural lesions?

**Please select only the best sequence(s); you do not need to select a sequence from each plane.**

### A.4 Joint space enhancement

View the JAMRIS-SIJ definitions for Inflammatory lesions. ◯ Hide ◯ View

Reference:

Herregods et al., Seminars in Arthritis and Rheumatism, 51 (2021) 1089-1098

**Table 1**
Scoring system for assessing inflammation of thesacroiliac joint by MRI according to the JAMRIS-SIJ scoring system proposed by the MRI in JIA working group of OMERACT. (current version, adapted and updated from reference [11]) (JAMRIS= Juvenile Idiopathic Arthritis MRI Score; SIJ= sacroiliac joint, MRI= Magnetic Resonance Imaging, JIA= Juvenile Idiopathic Arthritis, OMERACT= Outcome Measures in Rheumatology and Clinical Trials; BME= bone marrow edema, FS= fat suppressed).

| Item | Definition | Segmentation/slice | Score range/slice |
|---|---|---|---|
| Bone Marrow Edema (BME) | An ill defined area of high bone marrow signal intensity within the subchondral bone in the ilium or sacrum on fluid sensitive images | 4 quadrants/SIJ | 0–8 |
| BME Intensity | Presence of hyperintensity of the marrow on fluid sensitive images using the signal of the presacral veins or cerebrospinal fluid (CSF) as reference | 1 score /SIJ | 0–2 |
| BME Depth | Continuing increased signal on fluid sensitive images of depth ≥ 5mm/ ≥ 1cm from the articular surface | 1 score /SIJ | 0–2 |
| Osteitis | An ill-defined area of high bone marrow signal intensity within the subchondral bone in the ilium or sacrum on contrast enhanced T1 weighted sequences | 4 quadrants/SIJ | 0–8 |
| Joint Space Fluid | High signal intensity equivalent to the CSF on fluid sensitive sequences within the joint space of the cartilaginous portion of the SIJ | halves/SIJ | 0–4 |
| Joint Space Enhancement | Increased signal intensity on contrast enhanced T1 weighted sequences within the joint space of the cartilaginous portion of the SIJ | halves/SIJ | 0–4 |
| Inflammation in erosion cavity | Increased signal intensity on fluid sensitive or contrast enhanced T1 weighted sequences in an erosion cavity of the cartilaginous portion of the SIJ | halves/SIJ | 0–4 |
| Enthesitis | Increased signal intensity in bone marrow and/or adjacent soft tissue on fluid sensitive or contrast enhanced T1 weighted sequences at sites where ligaments and tendons attach to a bone excluding retroarticular enthesitis | score per case | 0–1 |
| Capsulitis | Increased signal on fluid sensitive or contrast enhanced T1 weighted sequences involving the superior portion of the SIJ capsule | superior halves/SIJ | 0–2 |

Statement of Overarching consideration for all definition: “in comparison to physiological changes normally seen in MRIs of age and sex matched children, and visible in two planes where available”

Pre-contrast sequences, small FOV

| | T1 w/o FS | T1 w/ FS | T1 Dixon | STIR | T2 w/o FS | T2 w/ FS | T2 Dixon | GRE |
|---|---|---|---|---|---|---|---|---|
| Coronal oblique | ☐ | ☐ | ☐ | ☐ | ☐ | ☐ | ☐ | ☐ |
| Axial oblique | ☐ | ☐ | ☐ | ☐ | ☐ | ☐ | ☐ | ☐ |

Other pre-contrast sequence(s):
Please specify FOV (whole pelvis or small FOV of SIJs), plane and sequence ________________

Post-contrast sequences, small FOV

| | T1 w/o FS | T1 w/ FS | T1 Dixon |
|---|---|---|---|
| Coronal oblique | ☐ | ☐ | ☐ |
| Axial oblique | ☐ | ☐ | ☐ |

Other post-contrast sequence(s):
Please specify FOV (whole pelvis or small FOV of SIJs), plane and sequence ________________

**Part 3: Which sequence(s) are best suited for evaluating the following (A.#) active and (B.#) structural lesions?**

**Please select only the best sequence(s); you do not need to select a sequence from each plane.**

## A.5 Inflammation in erosion cavity

View the JAMRIS-SIJ definitions for Inflammatory lesions. ◯ Hide ◯ View

Reference:

Herregods et al., Seminars in Arthritis and Rheumatism, 51 (2021) 1089-1098

**Table 1**
Scoring system for assessing inflammation of thesacroiliac joint by MRI according to the JAMRIS-SIJ scoring system proposed by the MRI in JIA working group of OMERACT. (current version, adapted and updated from reference [11]) (JAMRIS= Juvenile Idiopathic Arthritis MRI Score; SIJ= sacroiliac joint, MRI= Magnetic Resonance Imaging, JIA= Juvenile Idiopathic Arthritis, OMERACT= Outcome Measures in Rheumatology and Clinical Trials; BME= bone marrow edema, FS= fat suppressed).

| Item | Definition | Segmentation/slice | Score range/slice |
|---|---|---|---|
| Bone Marrow Edema (BME) | An ill defined area of high bone marrow signal intensity within the subchondral bone in the ilium or sacrum on fluid sensitive images | 4 quadrants/SIJ | 0–8 |
| BME Intensity | Presence of hyperintensity of the marrow on fluid sensitive images using the signal of the presacral veins or cerebrospinal fluid (CSF) as reference | 1 score /SIJ | 0–2 |
| BME Depth | Continuing increased signal on fluid sensitive images of depth ≥ 5mm/ ≥ 1cm from the articular surface | 1 score /SIJ | 0–2 |
| Osteitis | An ill-defined area of high bone marrow signal intensity within the subchondral bone in the ilium or sacrum on contrast enhanced T1 weighted sequences | 4 quadrants/SIJ | 0–8 |
| Joint Space Fluid | High signal intensity equivalent to the CSF on fluid sensitive sequences within the joint space of the cartilaginous portion of the SIJ | halves/SIJ | 0–4 |
| Joint Space Enhancement | Increased signal intensity on contrast enhanced T1 weighted sequences within the joint space of the cartilaginous portion of the SIJ | halves/SIJ | 0–4 |
| Inflammation in erosion cavity | Increased signal intensity on fluid sensitive or contrast enhanced T1 weighted sequences in an erosion cavity of the cartilaginous portion of the SIJ | halves/SIJ | 0–4 |
| Enthesitis | Increased signal intensity in bone marrow and/or adjacent soft tissue on fluid sensitive or contrast enhanced T1 weighted sequences at sites where ligaments and tendons attach to a bone excluding retroarticular enthesitis | score per case | 0–1 |
| Capsulitis | Increased signal on fluid sensitive or contrast enhanced T1 weighted sequences involving the superior portion of the SIJ capsule | superior halves/SIJ | 0–2 |

Statement of Overarching consideration for all definition: “in comparison to physiological changes normally seen in MRIs of age and sex matched children, and visible in two planes where available”

Pre-contrast sequences, small FOV

| | T1 w/o FS | T1 w/ FS | T1 Dixon | STIR | T2 w/o FS | T2 w/ FS | T2 Dixon | GRE |
|---|---|---|---|---|---|---|---|---|
| Coronal oblique | ☐ | ☐ | ☐ | ☐ | ☐ | ☐ | ☐ | ☐ |
| Axial oblique | ☐ | ☐ | ☐ | ☐ | ☐ | ☐ | ☐ | ☐ |

Other pre-contrast sequence(s):
Please specify FOV (whole pelvis or small FOV of SIJs), plane and sequence ________________

Post-contrast sequences, small FOV

| | T1 w/o FS | T1 w/ FS | T1 Dixon |
|---|---|---|---|
| Coronal oblique | ☐ | ☐ | ☐ |
| Axial oblique | ☐ | ☐ | ☐ |

Other post-contrast sequence(s):
Please specify FOV (whole pelvis or small FOV of SIJs), plane and sequence ________________

**Part 3: Which sequence(s) are best suited for evaluating the following (A.#) active and (B.#) structural lesions?**

**Please select only the best sequence(s); you do not need to select a sequence from each plane.**

## A.6 Enthesitis

View the JAMRIS-SIJ definitions for Inflammatory lesions. ◯ Hide ◯ View

Reference:

Herregods et al., Seminars in Arthritis and Rheumatism, 51 (2021) 1089-1098

**Table 1**
Scoring system for assessing inflammation of thesacroiliac joint by MRI according to the JAMRIS-SIJ scoring system proposed by the MRI in JIA working group of OMERACT. (current version, adapted and updated from reference [11]) (JAMRIS= Juvenile Idiopathic Arthritis MRI Score; SIJ= sacroiliac joint, MRI= Magnetic Resonance Imaging, JIA= Juvenile Idiopathic Arthritis, OMERACT= Outcome Measures in Rheumatology and Clinical Trials; BME= bone marrow edema, FS= fat suppressed).

| Item | Definition | Segmentation/slice | Score range/slice |
|---|---|---|---|
| Bone Marrow Edema (BME) | An ill defined area of high bone marrow signal intensity within the subchondral bone in the ilium or sacrum on fluid sensitive images | 4 quadrants/SIJ | 0–8 |
| BME Intensity | Presence of hyperintensity of the marrow on fluid sensitive images using the signal of the presacral veins or cerebrospinal fluid (CSF) as reference | 1 score /SIJ | 0–2 |
| BME Depth | Continuing increased signal on fluid sensitive images of depth ≥ 5mm/ ≥ 1cm from the articular surface | 1 score /SIJ | 0–2 |
| Osteitis | An ill-defined area of high bone marrow signal intensity within the subchondral bone in the ilium or sacrum on contrast enhanced T1 weighted sequences | 4 quadrants/SIJ | 0–8 |
| Joint Space Fluid | High signal intensity equivalent to the CSF on fluid sensitive sequences within the joint space of the cartilaginous portion of the SIJ | halves/SIJ | 0–4 |
| Joint Space Enhancement | Increased signal intensity on contrast enhanced T1 weighted sequences within the joint space of the cartilaginous portion of the SIJ | halves/SIJ | 0–4 |
| Inflammation in erosion cavity | Increased signal intensity on fluid sensitive or contrast enhanced T1 weighted sequences in an erosion cavity of the cartilaginous portion of the SIJ | halves/SIJ | 0–4 |
| Enthesitis | Increased signal intensity in bone marrow and/or adjacent soft tissue on fluid sensitive or contrast enhanced T1 weighted sequences at sites where ligaments and tendons attach to a bone excluding retroarticular enthesitis | score per case | 0–1 |
| Capsulitis | Increased signal on fluid sensitive or contrast enhanced T1 weighted sequences involving the superior portion of the SIJ capsule | superior halves/SIJ | 0–2 |

Statement of Overarching consideration for all definition: "in comparison to physiological changes normally seen in MRIs of age and sex matched children, and visible in two planes where available"

Pre-contrast sequences, small FOV

| | T1 w/o FS | T1 w/ FS | T1 Dixon | STIR | T2 w/o FS | T2 w/ FS | T2 Dixon | GRE |
|---|---|---|---|---|---|---|---|---|
| Coronal oblique | ☐ | ☐ | ☐ | ☐ | ☐ | ☐ | ☐ | ☐ |
| Axial oblique | ☐ | ☐ | ☐ | ☐ | ☐ | ☐ | ☐ | ☐ |

Other pre-contrast sequence(s):
Please specify FOV (whole pelvis or small FOV of SIJs), plane and sequence ________________

Post-contrast sequences, small FOV

| | T1 w/o FS | T1 w/ FS | T1 Dixon |
|---|---|---|---|
| Coronal oblique | ☐ | ☐ | ☐ |
| Axial oblique | ☐ | ☐ | ☐ |

Other post-contrast sequence(s):
Please specify FOV (whole pelvis or small FOV of SIJs), plane and sequence ________________

## Part 3: Which sequence(s) are best suited for evaluating the following (A.#) active and (B.#) structural lesions?

**Please select only the best sequence(s); you do not need to select a sequence from each plane.**

### A.7 Capsulitis

View the JAMRIS-SIJ definitions for Inflammatory lesions. ◯ Hide ◯ View

Reference:

Herregods et al., Seminars in Arthritis and Rheumatism, 51 (2021) 1089-1098

**Table 1**
Scoring system for assessing inflammation of thesacroiliac joint by MRI according to the JAMRIS-SIJ scoring system proposed by the MRI in JIA working group of OMERACT. (current version, adapted and updated from reference [11]) (JAMRIS= Juvenile Idiopathic Arthritis MRI Score; SIJ= sacroiliac joint, MRI= Magnetic Resonance Imaging, JIA= Juvenile Idiopathic Arthritis, OMERACT= Outcome Measures in Rheumatology and Clinical Trials; BME= bone marrow edema, FS= fat suppressed).

| Item | Definition | Segmentation/slice | Score range/slice |
|---|---|---|---|
| Bone Marrow Edema (BME) | An ill defined area of high bone marrow signal intensity within the subchondral bone in the ilium or sacrum on fluid sensitive images | 4 quadrants/SIJ | 0–8 |
| BME Intensity | Presence of hyperintensity of the marrow on fluid sensitive images using the signal of the presacral veins or cerebrospinal fluid (CSF) as reference | 1 score /SIJ | 0–2 |
| BME Depth | Continuing increased signal on fluid sensitive images of depth ≥ 5mm/ ≥ 1cm from the articular surface | 1 score /SIJ | 0–2 |
| Osteitis | An ill-defined area of high bone marrow signal intensity within the subchondral bone in the ilium or sacrum on contrast enhanced T1 weighted sequences | 4 quadrants/SIJ | 0–8 |
| Joint Space Fluid | High signal intensity equivalent to the CSF on fluid sensitive sequences within the joint space of the cartilaginous portion of the SIJ | halves/SIJ | 0–4 |
| Joint Space Enhancement | Increased signal intensity on contrast enhanced T1 weighted sequences within the joint space of the cartilaginous portion of the SIJ | halves/SIJ | 0–4 |
| Inflammation in erosion cavity | Increased signal intensity on fluid sensitive or contrast enhanced T1 weighted sequences in an erosion cavity of the cartilaginous portion of the SIJ | halves/SIJ | 0–4 |
| Enthesitis | Increased signal intensity in bone marrow and/or adjacent soft tissue on fluid sensitive or contrast enhanced T1 weighted sequences at sites where ligaments and tendons attach to a bone excluding retroarticular enthesitis | score per case | 0–1 |
| Capsulitis | Increased signal on fluid sensitive or contrast enhanced T1 weighted sequences involving the superior portion of the SIJ capsule | superior halves/SIJ | 0–2 |

Statement of Overarching consideration for all definition: “in comparison to physiological changes normally seen in MRIs of age and sex matched children, and visible in two planes where available”

Pre-contrast sequences, small FOV

| | T1 w/o FS | T1 w/ FS | T1 Dixon | STIR | T2 w/o FS | T2 w/ FS | T2 Dixon | GRE |
|---|---|---|---|---|---|---|---|---|
| Coronal oblique | ☐ | ☐ | ☐ | ☐ | ☐ | ☐ | ☐ | ☐ |
| Axial oblique | ☐ | ☐ | ☐ | ☐ | ☐ | ☐ | ☐ | ☐ |

Other pre-contrast sequence(s):
Please specify FOV (whole pelvis or small FOV of SIJs), plane and sequence ________________

Post-contrast sequences, small FOV

| | T1 w/o FS | T1 w/ FS | T1 Dixon |
|---|---|---|---|
| Coronal oblique | ☐ | ☐ | ☐ |
| Axial oblique | ☐ | ☐ | ☐ |

Other post-contrast sequence(s):
Please specify FOV (whole pelvis or small FOV of SIJs), plane and sequence ________________

**Part 3: Which sequence(s) are best suited for evaluating the following (A.#) active and (B.#) structural lesions?**

**Please select only the best sequence(s); you do not need to select a sequence from each plane.**

## B.1 Sclerosis

View the JAMRIS-SIJ definitions for structural lesions. ◯ Hide ◯ View

Reference:

Herregods et al., Seminars in Arthritis and Rheumatism, 51 (2021) 1099-1107

**Table 1**
Scoring system for assessing structural damage of the sacroiliac joint by MRI according to the JAMRIS-SIJ scoring system proposed by the MRI in JIA working group of OMERACT. (current version, adapted and updated from reference [11]) (JAMRIS= Juvenile Idiopathic Arthritis MRI Score; SIJ= sacroiliac joint, MRI= Magnetic Resonance Imaging, JIA= Juvenile Idiopathic Arthritis, OMERACT= Outcome Measures in Rheumatology and Clinical Trials; BME= bone marrow edema, FS= fat suppressed).

| Item | Definition | Segmentation/slice | Score range/slice |
|---|---|---|---|
| Sclerosis | A substantially wider than normal area of very low bone marrow signal intensity within the subchondral bone in the ilium or sacrum on a non-fat suppressed sequence, preferably a non-fat suppressed T1 weighted sequence. This feature must also be present on all other sequences, as available | 4 quadrants/SIJ | 0-8 |
| Erosions | A focal loss of the low signal of cortical bone at the osteochondral interface and adjacent marrow matrix on T1 weighted images | 4 quadrants/SIJ | 0-8 |
| Fat lesion (fat metaplasia) | Homogeneous increased signal intensity within the subchondral bone marrow on T1 weighted images | 4 quadrants/SIJ | 0-8 |
| Backfill | A high signal on non-contrast enhanced T1 weighted sequences in a typical location for an erosion, with signal intensity greater than normal bone marrow, clearly demarcated from adjacent bone marrow by an irregular band of low signal reflecting sclerosis at the border of the original erosion | halves/SIJ | 0-4 |
| Ankylosis | Presence of signal equivalent to regional bone marrow continuously bridging a portion of the joint space between the iliac and sacral bones | halves/SIJ | 0-4 |

Pre-contrast sequences, small FOV

| | T1 w/o FS | T1 w/ FS | T1 Dixon | STIR | T2 w/o FS | T2 w/ FS | T2 Dixon | GRE |
|---|---|---|---|---|---|---|---|---|
| Coronal oblique | ☐ | ☐ | ☐ | ☐ | ☐ | ☐ | ☐ | ☐ |
| Axial oblique | ☐ | ☐ | ☐ | ☐ | ☐ | ☐ | ☐ | ☐ |

Other pre-contrast sequence(s):
Please specify FOV (whole pelvis or small FOV of SIJs), plane and sequence ________________

Post-contrast sequences, small FOV

| | T1 w/o FS | T1 w/ FS | T1 Dixon |
|---|---|---|---|
| Coronal oblique | ☐ | ☐ | ☐ |
| Axial oblique | ☐ | ☐ | ☐ |

Other post-contrast sequence(s):
Please specify FOV (whole pelvis or small FOV of SIJs), plane and sequence ________________

**Part 3: Which sequence(s) are best suited for evaluating the following (A.#) active and (B.#) structural lesions?**

**Please select only the best sequence(s); you do not need to select a sequence from each plane.**

## B.2 Erosions

View the JAMRIS-SIJ definitions for structural lesions. ◯ Hide ◯ View

Reference:

Herregods et al., Seminars in Arthritis and Rheumatism, 51 (2021) 1099-1107

**Table 1**
Scoring system for assessing structural damage of the sacroiliac joint by MRI according to the JAMRIS-SIJ scoring system proposed by the MRI in JIA working group of OMERACT. (current version, adapted and updated from reference [11]) (JAMRIS= Juvenile Idiopathic Arthritis MRI Score; SIJ= sacroiliac joint, MRI= Magnetic Resonance Imaging, JIA= Juvenile Idiopathic Arthritis, OMERACT= Outcome Measures in Rheumatology and Clinical Trials; BME= bone marrow edema, FS= fat suppressed).

| Item | Definition | Segmentation/slice | Score range/slice |
|---|---|---|---|
| Sclerosis | A substantially wider than normal area of very low bone marrow signal intensity within the subchondral bone in the ilium or sacrum on a non-fat suppressed sequence, preferably a non-fat suppressed T1 weighted sequence. This feature must also be present on all other sequences, as available | 4 quadrants/SIJ | 0-8 |
| Erosions | A focal loss of the low signal of cortical bone at the osteochondral interface and adjacent marrow matrix on T1 weighted images | 4 quadrants/SIJ | 0-8 |
| Fat lesion (fat metaplasia) | Homogeneous increased signal intensity within the subchondral bone marrow on T1 weighted images | 4 quadrants/SIJ | 0-8 |
| Backfill | A high signal on non-contrast enhanced T1 weighted sequences in a typical location for an erosion, with signal intensity greater than normal bone marrow, clearly demarcated from adjacent bone marrow by an irregular band of low signal reflecting sclerosis at the border of the original erosion | halves/SIJ | 0-4 |
| Ankylosis | Presence of signal equivalent to regional bone marrow continuously bridging a portion of the joint space between the iliac and sacral bones | halves/SIJ | 0-4 |

Pre-contrast sequences, small FOV

| | T1 w/o FS | T1 w/ FS | T1 Dixon | STIR | T2 w/o FS | T2 w/ FS | T2 Dixon | GRE |
|---|---|---|---|---|---|---|---|---|
| Coronal oblique | ☐ | ☐ | ☐ | ☐ | ☐ | ☐ | ☐ | ☐ |
| Axial oblique | ☐ | ☐ | ☐ | ☐ | ☐ | ☐ | ☐ | ☐ |

Other pre-contrast sequence(s):
Please specify FOV (whole pelvis or small FOV of SIJs), plane and sequence ________________

Post-contrast sequences, small FOV

| | T1 w/o FS | T1 w/ FS | T1 Dixon |
|---|---|---|---|
| Coronal oblique | ☐ | ☐ | ☐ |
| Axial oblique | ☐ | ☐ | ☐ |

Other post-contrast sequence(s):
Please specify FOV (whole pelvis or small FOV of SIJs), plane and sequence ________________

## Part 3: Which sequence(s) are best suited for evaluating the following (A.#) active and (B.#) structural lesions?

**Please select only the best sequence(s); you do not need to select a sequence from each plane.**

### B.3 Fat lesion (fat metaplasia)

View the JAMRIS-SIJ definitions for structural lesions. ○ Hide ○ View

Reference:

Herregods et al., Seminars in Arthritis and Rheumatism, 51 (2021) 1099-1107

**Table 1**
Scoring system for assessing structural damage of the sacroiliac joint by MRI according to the JAMRIS-SIJ scoring system proposed by the MRI in JIA working group of OMERACT. (current version, adapted and updated from reference [11]) (JAMRIS= Juvenile Idiopathic Arthritis MRI Score; SIJ= sacroiliac joint, MRI= Magnetic Resonance Imaging, JIA= Juvenile Idiopathic Arthritis, OMERACT= Outcome Measures in Rheumatology and Clinical Trials; BME= bone marrow edema, FS= fat suppressed).

| Item | Definition | Segmentation/slice | Score range/slice |
|---|---|---|---|
| Sclerosis | A substantially wider than normal area of very low bone marrow signal intensity within the subchondral bone in the ilium or sacrum on a non-fat suppressed sequence, preferably a non-fat suppressed T1 weighted sequence. This feature must also be present on all other sequences, as available | 4 quadrants/SIJ | 0-8 |
| Erosions | A focal loss of the low signal of cortical bone at the osteochondral interface and adjacent marrow matrix on T1 weighted images | 4 quadrants/SIJ | 0-8 |
| Fat lesion (fat metaplasia) | Homogeneous increased signal intensity within the subchondral bone marrow on T1 weighted images | 4 quadrants/SIJ | 0-8 |
| Backfill | A high signal on non-contrast enhanced T1 weighted sequences in a typical location for an erosion, with signal intensity greater than normal bone marrow, clearly demarcated from adjacent bone marrow by an irregular band of low signal reflecting sclerosis at the border of the original erosion | halves/SIJ | 0-4 |
| Ankylosis | Presence of signal equivalent to regional bone marrow continuously bridging a portion of the joint space between the iliac and sacral bones | halves/SIJ | 0-4 |

Pre-contrast sequences, small FOV

| | T1 w/o FS | T1 w/ FS | T1 Dixon | STIR | T2 w/o FS | T2 w/ FS | T2 Dixon | GRE |
|---|---|---|---|---|---|---|---|---|
| Coronal oblique | ☐ | ☐ | ☐ | ☐ | ☐ | ☐ | ☐ | ☐ |
| Axial oblique | ☐ | ☐ | ☐ | ☐ | ☐ | ☐ | ☐ | ☐ |

Other pre-contrast sequence(s):
Please specify FOV (whole pelvis or small FOV of SIJs), plane and sequence ________________

Post-contrast sequences, small FOV

| | T1 w/o FS | T1 w/ FS | T1 Dixon |
|---|---|---|---|
| Coronal oblique | ☐ | ☐ | ☐ |
| Axial oblique | ☐ | ☐ | ☐ |

Other post-contrast sequence(s):
Please specify FOV (whole pelvis or small FOV of SIJs), plane and sequence ________________

**Part 3: Which sequence(s) are best suited for evaluating the following (A.#) active and (B.#) structural lesions?**

**Please select only the best sequence(s); you do not need to select a sequence from each plane.**

## B.4 Backfill

View the JAMRIS-SIJ definitions for structural lesions. ◯ Hide ◯ View

Reference:

Herregods et al., Seminars in Arthritis and Rheumatism, 51 (2021) 1099-1107

**Table 1**
Scoring system for assessing structural damage of the sacroiliac joint by MRI according to the JAMRIS-SIJ scoring system proposed by the MRI in JIA working group of OMERACT. (current version, adapted and updated from reference [11]) (JAMRIS= Juvenile Idiopathic Arthritis MRI Score; SIJ= sacroiliac joint, MRI= Magnetic Resonance Imaging, JIA= Juvenile Idiopathic Arthritis, OMERACT= Outcome Measures in Rheumatology and Clinical Trials; BME= bone marrow edema, FS= fat suppressed).

| Item | Definition | Segmentation/slice | Score range/slice |
|---|---|---|---|
| Sclerosis | A substantially wider than normal area of very low bone marrow signal intensity within the subchondral bone in the ilium or sacrum on a non-fat suppressed sequence, preferably a non-fat suppressed T1 weighted sequence. This feature must also be present on all other sequences, as available | 4 quadrants/SIJ | 0-8 |
| Erosions | A focal loss of the low signal of cortical bone at the osteochondral interface and adjacent marrow matrix on T1 weighted images | 4 quadrants/SIJ | 0-8 |
| Fat lesion (fat metaplasia) | Homogeneous increased signal intensity within the subchondral bone marrow on T1 weighted images | 4 quadrants/SIJ | 0-8 |
| Backfill | A high signal on non-contrast enhanced T1 weighted sequences in a typical location for an erosion, with signal intensity greater than normal bone marrow, clearly demarcated from adjacent bone marrow by an irregular band of low signal reflecting sclerosis at the border of the original erosion | halves/SIJ | 0-4 |
| Ankylosis | Presence of signal equivalent to regional bone marrow continuously bridging a portion of the joint space between the iliac and sacral bones | halves/SIJ | 0-4 |

Pre-contrast sequences, small FOV

| | T1 w/o FS | T1 w/ FS | T1 Dixon | STIR | T2 w/o FS | T2 w/ FS | T2 Dixon | GRE |
|---|---|---|---|---|---|---|---|---|
| Coronal oblique | ☐ | ☐ | ☐ | ☐ | ☐ | ☐ | ☐ | ☐ |
| Axial oblique | ☐ | ☐ | ☐ | ☐ | ☐ | ☐ | ☐ | ☐ |

Other pre-contrast sequence(s):
Please specify FOV (whole pelvis or small FOV of SIJs), plane and sequence ____________________

Post-contrast sequences, small FOV

| | T1 w/o FS | T1 w/ FS | T1 Dixon |
|---|---|---|---|
| Coronal oblique | ☐ | ☐ | ☐ |
| Axial oblique | ☐ | ☐ | ☐ |

Other post-contrast sequence(s):
Please specify FOV (whole pelvis or small FOV of SIJs), plane and sequence ____________________

**Part 3: Which sequence(s) are best suited for evaluating the following (A.#) active and (B.#) structural lesions?**

**Please select only the best sequence(s); you do not need to select a sequence from each plane.**

## B.5 Ankylosis

View the JAMRIS-SIJ definitions for structural lesions. ○ Hide ○ View

Reference:

Herregods et al., Seminars in Arthritis and Rheumatism, 51 (2021) 1099-1107

**Table 1**
Scoring system for assessing structural damage of the sacroiliac joint by MRI according to the JAMRIS-SIJ scoring system proposed by the MRI in JIA working group of OMERACT. (current version, adapted and updated from reference [11]) (JAMRIS= Juvenile Idiopathic Arthritis MRI Score; SIJ= sacroiliac joint, MRI= Magnetic Resonance Imaging, JIA= Juvenile Idiopathic Arthritis, OMERACT= Outcome Measures in Rheumatology and Clinical Trials; BME= bone marrow edema, FS= fat suppressed).

| Item | Definition | Segmentation/slice | Score range/slice |
|---|---|---|---|
| Sclerosis | A substantially wider than normal area of very low bone marrow signal intensity within the subchondral bone in the ilium or sacrum on a non-fat suppressed sequence, preferably a non-fat suppressed T1 weighted sequence. This feature must also be present on all other sequences, as available | 4 quadrants/SIJ | 0-8 |
| Erosions | A focal loss of the low signal of cortical bone at the osteochondral interface and adjacent marrow matrix on T1 weighted images | 4 quadrants/SIJ | 0-8 |
| Fat lesion (fat metaplasia) | Homogeneous increased signal intensity within the subchondral bone marrow on T1 weighted images | 4 quadrants/SIJ | 0-8 |
| Backfill | A high signal on non-contrast enhanced T1 weighted sequences in a typical location for an erosion, with signal intensity greater than normal bone marrow, clearly demarcated from adjacent bone marrow by an irregular band of low signal reflecting sclerosis at the border of the original erosion | halves/SIJ | 0-4 |
| Ankylosis | Presence of signal equivalent to regional bone marrow continuously bridging a portion of the joint space between the iliac and sacral bones | halves/SIJ | 0-4 |

Pre-contrast sequences, small FOV

| | T1 w/o FS | T1 w/ FS | T1 Dixon | STIR | T2 w/o FS | T2 w/ FS | T2 Dixon | GRE |
|---|---|---|---|---|---|---|---|---|
| Coronal oblique | ☐ | ☐ | ☐ | ☐ | ☐ | ☐ | ☐ | ☐ |
| Axial oblique | ☐ | ☐ | ☐ | ☐ | ☐ | ☐ | ☐ | ☐ |

Other pre-contrast sequence(s):
Please specify FOV (whole pelvis or small FOV of SIJs), plane and sequence ____________________

Post-contrast sequences, small FOV

| | T1 w/o FS | T1 w/ FS | T1 Dixon |
|---|---|---|---|
| Coronal oblique | ☐ | ☐ | ☐ |
| Axial oblique | ☐ | ☐ | ☐ |

Other post-contrast sequence(s):
Please specify FOV (whole pelvis or small FOV of SIJs), plane and sequence ____________________

## Selection of sequences

List of sequences you had selected as essential on page 2:

[p2q3]

If you would like to make any changes to your list of essential sequences, please enter your revised version here: ______________________

List of sequences you had selected as optional on page 2:

[p2q4]

If you would like to make any changes to your list of optional sequences, please enter your revised version here: ______________________

## End of Survey

Thank you very much for completing this survey!
Please add below if you have any other comments.
